# Hypoxia Microenvironment Preconditioning Attenuated Myocardial Ischemia‐Reperfusion Injury via Stc1‐Mediating Cardiomyocyte Self‐Protection and Neutrophil Polarization

**DOI:** 10.1002/advs.202411880

**Published:** 2024-12-16

**Authors:** Haoxiang Huang, Yifei Ruan, Chuling Li, Hao Zheng, Yating Tang, Yijin Chen, Fengling He, Yu Liu, Guangkai Wu, Zhenhua Li, Yuegang Wang, Yulin Liao, Jianping Bin, Yanmei Chen

**Affiliations:** ^1^ Department of Cardiology State Key Laboratory of Organ Failure Research Nanfang Hospital Southern Medical University 1838 North Guangzhou Avenue Guangzhou 510515 China; ^2^ Guangdong Provincial Key Laboratory of Cardiac Function and Microcirculation Guangzhou 510515 China; ^3^ The Tenth Affiliated Hospital of Southern Medical University Dongguan Guangdong 523059 China

**Keywords:** ischemia/reperfusion injury, ischemic preconditioning therapy, microenvironment, secretome, stanniocalcin 1

## Abstract

Ischemic preconditioning (IPC) therapy application to attenuate myocardial ischemia‐reperfusion (MI/R) injury in clinical practice remains challenging. The secretome, derived from hypoxia‐preconditioned cardiomyocytes (SHPC), potentially mimics the IPC microenvironment and facilitates IPC clinical translation. This study aims to determine whether SHPC can be a feasible alternative to IPC for attenuating MI/R injury, and to identify the functional factor of SHPC. The ultrafiltration technique is applied to generate an SHPC formulation that is intramyocardially injected before reperfusion in a murine MI/R model. The effects of SHPC on cardiomyocyte apoptosis, pyroptosis, and neutrophil polarization are evaluated. Secretomics, neutralizing antibodies, and recombinant proteins are employed to identify the functional factor in SHPC. Co‐immunoprecipitation assays, RNA sequencing, and site‐directed mutagenesis are conducted to investigate the underlying mechanism. Additionally, a recombinant functional factor‐encapsulated hydrogel is developed for intrapericardial injections (iPC). An intramyocardial SHPC injection in MI/R‐injured mice strikingly reduces infarct size and the expression of cardiac injury biomarker while improving cardiac function. SHPC eliminated mitochondrial reactive oxygen species and triggered neutrophil polarization to reduce cardiomyocyte apoptosis/pyroptosis upon hypoxia/reoxygenation injury. Stanniocalcin 1 (Stc1) is identified as the functional factor in SHPC, mediating hypoxic microenvironment. Mechanistically, hypoxia‐preconditioned cardiomyocytes secrete Stc1 into the microenvironment and activate calcium‐sensing receptor (CaSR) that increases Stat3 phosphorylation at Ser727 via nitric oxide synthase 2 (NOS2)‐mediated S‐nitrosylation, thereby decreasing cardiomyocyte apoptosis/pyroptosis in an autocrine mechanism. Simultaneously, Stc1 facilitates cardiomyocyte‐neutrophil crosstalk, thereby triggering neutrophil polarization to reduce inflammatory damage via the CaSR/NOS2/Stat3 axis in a paracrine mechanism. Pericardial delivery of a recombinant rStc1‐encapsulated hydrogel has extended the therapeutic time window of rStc1, improving long‐term cardiac function. The hypoxia microenvironment preconditioning, which mimicked by SHPC, attenuated MI/R injury via Stc1‐mediated cardiomyocyte self‐protection and neutrophil polarization. This study suggests that SHPC, with hypoxia preconditioning factor Stc1, represents a clinically feasible alternative to IPC for attenuating MI/R injury.

## Graphical abstract

The IPC microenvironment mimicked by SHPC attenuates MI/R injury, which is attributed to Stc1‐mediated dual mechanisms that improve cardiomyocyte self‐protection and promote neutrophil polarization via the CaSR/NOS2/Stat3 pathway. Pericardial delivery of rStc1‐encapsulated hydrogels extends the therapeutic time window of Stc1 and improves long‐term cardiac function. The results indicate SHPC with hypoxia preconditioning factor Stc1 as a clinically feasible alternative to IPC for attenuating MI/R injury.

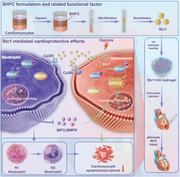

## 1 Introduction

Myocardial ischemia‐reperfusion (MI/R) injury is a common complication in patients receiving revascularization therapy for myocardial infarction, frequently causing myocardial stunning, reperfusion arrhythmia, myocardial infarct size expansion, and heart failure.^[^ ^1^ ^]^ Ischemic preconditioning (IPC) therapy is pre‐exposure to ischemia to develop tolerance to the deleterious effects of prolonged MI/R injury.^[^ ^2^, ^3^ ^]^ Many studies have established the therapeutic value of IPC in alleviating MI/R injury, while its routine clinical use remains challenging. This is primarily because IPC treatment must be applied before long‐duration ischemia occurs while myocardial infarction events remain unpredictable.^[^ ^4^ ^]^

An increasing number of studies have focused on key mechanisms that mediate endogenous cardio‐protection to facilitate the clinical translation of IPC therapy.^[^ ^5^, ^6^ ^]^ Understanding the components and intercellular interactions within the microenvironment is crucial for recognizing the endogenous mechanisms of IPC. The secretome, which contains a cocktail of bioactive factors, hormones, extracellular vesicles, proteins, and other metabolites secreted by cells, is the most important component of the microenvironment, and is fundamental for cellular communication.^[^ ^7^ ^]^ Recently, secretome therapy has emerged as a promising cell‐free alternative therapy, because it has the advantage of being available at any time compared to the cell product itself and overcoming various potential adverse effects of traditional cell therapy. Previous studies have revealed the effectiveness and safety of mesenchymal stem cell (MSC) secretome application in promoting tissue repair and regulating immune response. Hypoxia‐elicited MSC‐derived exosomes have facilitated cardiac repair through multiple miRNA‐mediated mechanisms that prevent cardiomyocyte death in myocardial infarction.^[^ ^8^, ^9^ ^]^ The cardiomyocyte secretome directly interacts with membrane receptors to exhibit its functions through autocrine and paracrine mechanisms in the microenvironment. The secretome derived from hypoxia‐preconditioned cardiomyocytes (SHPC) more effectively mimics the microenvironment of IPC, because it exerts a dual therapeutic effect via autocrine and paracrine means compared to those derived from hypoxia‐preconditioned MSCs. Previous studies have revealed that stressed cardiomyocytes secrete key proteins, such as GRP94, GRP78, and CRT, to decrease MI/R injury and increase tissue recovery in an autocrine mechanism.^[^ ^10^ ^]^ Meanwhile, normal cardiomyocytes secrete some factors to communicate with other cells in a paracrine mechanism.^[^ ^11^ ^]^ Based on these significant implications, it is plausible that SHPC may mimic the microenvironment of IPC and produce the cardioprotective effect.

Accordingly, we hypothesized that hypoxic microenvironment preconditioning, mimicked by SHPC, attenuated MI/R injury, thereby facilitating the clinical translation of IPC therapy. We used the ultrafiltration technique to generate an SHPC formulation and investigated the effects of SHPC on cardiac function in an MI/R mouse model. Besides, we identified the functional factor in SHPC and investigated the potential mechanism using secretomics analysis, neutralizing antibodies, and recombinant proteins. Our result indicated that SHPC with hypoxia preconditioning factor Stc1 may present a promising alternative to IPC therapy for alleviating MI/R injury.

## 2 Results

### 2.1 The SHPC Attenuated MI/R Injury

**Figure 1:**
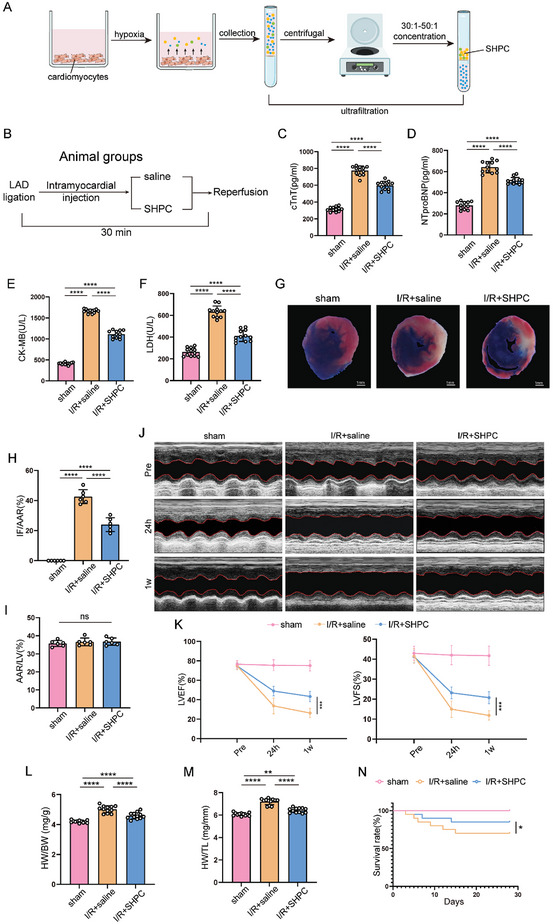
The secretome derived from hypoxia‐preconditioned cardiomyocytes (SHPC) attenuated myocardial ischemia‐reperfusion (MI/R) injury. A) Scheme depicting SHPC extraction. B) Treatment groups. C–F) Circulating cardiac troponin T (cTnT), N‐terminal pro‐B‐type natriuretic peptide (NTpro‐BNP), creatine kinase‐MB (CK‐MB), and lactate dehydrogenase (LDH) levels in mice (n = 12 mice) with saline or SHPC injection 24 h after I/R surgery measured by ELISA kits. G–I) Representative Evans blue and TTC‐stained heart sections, and quantitative data from mice with saline or SHPC injection 24 h after I/R surgery. IF: infarct area, AAR: area at risk, LV: left ventricle (scale bar = 1 mm; n = 6 mice). J,K) Cardiac function analyzed using echocardiography at pre‐I/R surgery, 24 h, and 1 week post‐I/R surgery time points (n = 6 mice). L,M) The heart weight (HW)/body weight (BW) ratio and HW/tibial length (TL) ratio at 4 weeks after I/R surgery (n = 12 mice). N) Four‐week survival curves of mice receiving saline or SHPC injection (n = 20 mice). Statistical significance was calculated using one‐way ANOVA in (C–F), (H–I), and (L–M) and two‐way ANOVA in (K) and the log‐rank (Mantel–Cox) test in N; *P < 0.05, **P < 0.01, ****P < 0.0001.

To obtain the hypoxia preconditioned secretome, neonatal cardiomyocytes were cultured under hypoxic conditions for 12 hours, and the hypoxia supernatant was then collected and concentrated by ultrafiltration to generate the SHPC formulation (**Figure** **1**A). Mice underwent LAD occlusion for 30 minutes, followed by 24 hours of reperfusion to generate the MI/R model (Figure 1B). The SHPC was injected intramyocardially before reperfusion to assess its effect on MI/R injury during ligation. Figure 1C–F illustrated that SHPC attenuated MI/R injury, as detected by cardiac injury biomarkers of cardiac troponin T (cTnT), N‐terminal pro‐B‐type natriuretic peptide (NTpro‐BNP), creatine kinase‐MB (CK‐MB), and lactate dehydrogenase (LDH) in the plasma. Intramyocardial SHPC injection strikingly decreased the infarct size in the area at risk at 24 hours post‐MI/R as compared with untreated MI/R mice (Figure 1G–I). SHPC markedly improved the cardiac contractile function at 24 hours and 1 week post‐MI/R, as reflected by an increase in the left ventricular ejection fraction (Figure 1J,K), and fractional shortening (Figure 1J,K). Additionally, SHPC reduces the heart weight (HW)/body weight (BW) ratio and HW/tibial length (TL) ratio at 4 weeks post‐MI/R (Figure 1L,M). As shown in Figure 1N, SHPC‐treated MI/R mice demonstrated a significantly lower mortality rate than untreated MI/R mice at 28 days. Overall, these data indicate that SHPC attenuated cardiac I/R injury.

### 2.2 SHPC Attenuated MI/R Injury by Reducing Cardiomyocyte Apoptosis/Pyroptosis and Promoting Neutrophil Polarization

**Figure 2:**
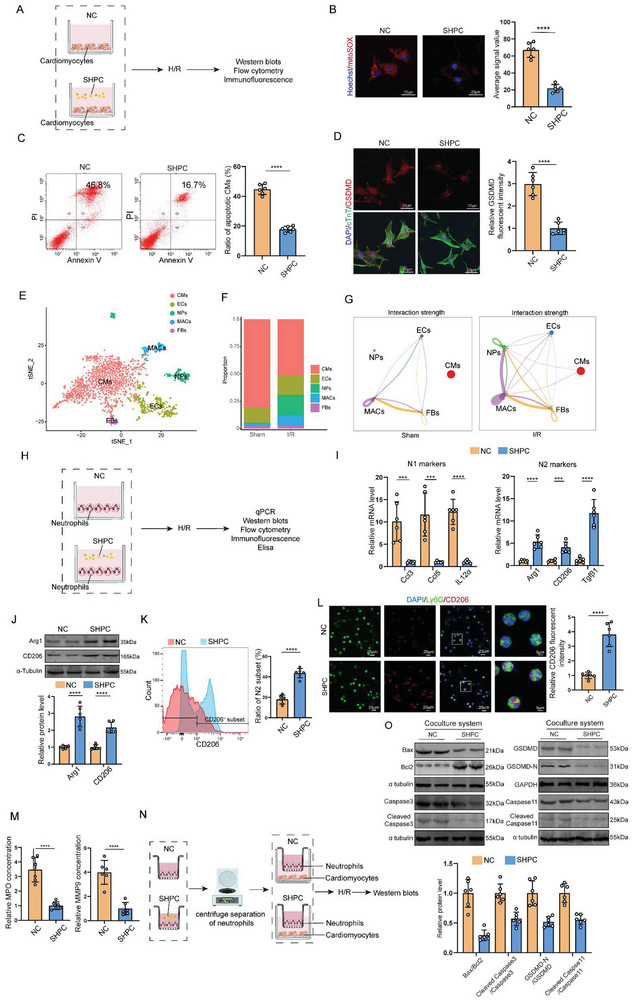
SHPC attenuated MI/R injury via cardiomyocyte apoptosis/pyroptosis and neutrophil polarization modulation. A) Scheme depicting SHPC to treat cardiomyocytes. B) Mitochondria‐derived ROS staining in NC‐ or SHPC‐treated cardiomyocytes after H/R treatment (176 cardiomyocytes from 6 mice in the NC group and 157 cardiomyocytes from 6 mice in the SHPC group; scale bar = 20 µm). C) Flow cytometry analysis of NC‐ or SHPC‐treated cardiomyocyte apoptosis after H/R treatment. Apoptotic cells include Annexin V(+)/PI(−) and Annexin V(+)/PI(+) cells (n = 6 cell samples). D) Immunofluorescent staining of GSDMD (red) and cTnT (green) in NC‐ or SHPC‐treated cardiomyocytes after H/R treatment (289 cardiomyocytes from 6 mice in the NC group and 317 cardiomyocytes from 6 mice in the SHPC group; scale bar = 20 µm). E) Cell grouping of sham and I/R heart tissues from single‐cell RNA sequencing (scRNA‐seq) data. F) Quantitative changes in different cell types during I/R from single‐cell RNA sequencing (scRNA‐seq) data. G) scRNA‐seq data predicting cellular communication between different cells in the heart under sham and I/R states. H) Scheme depicting SHPC extraction to treat neutrophils. I) N1 and N2 marker mRNA levels in NC‐ or SHPC‐treated neutrophils after H/R treatment (n = 6 cell samples). J) NC‐ or SHPC‐treated neutrophils collected for immunoblotting for Arg1 and CD206 expression after H/R treatment (n = 6 cell samples). K) NC‐ or SHPC‐treated neutrophils collected for flow cytometry of CD206‐positive cells after H/R treatment (n = 6 cell samples). L) NC‐ or SHPC‐treated neutrophils collected for immunofluorescent staining for Ly6G (green) and CD206 (red) after H/R treatment (426 neutrophils from 6 mice in the NC group and 396 neutrophils from 6 mice in the SHPC group; scale bar = 20 µm [three on the left] and 5 µm [rightmost]). M) ELISA results indicating MPO and MMP9 concentrations in NC‐ or SHPC‐treated neutrophil supernatants after H/R treatment (n = 6 supernatant samples). N) Scheme illustrating cell co‐culture system of neutrophils and cardiomyocytes. O) Bax, Bcl2, Cleaved caspase 3, Caspase 3, Cleaved GSDMD‐N, GSDMD, Cleaved caspase 11, and Caspase11 protein levels in cardiomyocytes co‐cultured with NC‐ or SHPC‐treated neutrophils (n = 6 cell samples). Statistical significance was calculated with an unpaired *t*‐test in (B–D), (I–M), and (O). ***P < 0.001 and ****P < 0.0001.

To determine whether SHPC attenuates MI/R injury via reducing cardiomyocyte death, cardiomyocytes were cultured with the SHPC before undergoing hypoxia/reoxygenation (H/R) injury (**Figure** **2**A). The SHPC treatment reduced cardiomyocyte death as detected by propidium iodide (PI) staining (Figure S1A, Supporting Information). SHPC treatment significantly reduced overall ROS and mitochondrial ROS (mtROS) level of cardiomyocytes (Figure 2B; Figure S1B, Supporting Information). Specifically, SHPC treatment reduced cardiomyocyte apoptosis and pyroptosis as indicated by the mRNA level of Bax, Bcl2, GSDMD, and Caspase11, whereas ferroptosis and necrosis marker levels were not affected (Figure S1C, Supporting Information). Additionally, Western blotting revealed that SHPC treatment decreased the Bax/Bcl2 and Cleaved Caspase3/Caspase3 ratio as well as the Cleaved GSDMD‐N/GSDMD and Cleaved Caspase11/Caspase11 ratio (Figure S1D, Supporting Information), indicating that SHPC reduced cardiomyocyte apoptosis and pyroptosis. Annexin V‐FITC/PI flow cytometry, TUNEL staining and immunofluorescent staining further confirmed reduced cardiomyocyte apoptosis and pyroptosis in SHPC treatment (Figure 2C,D; Figure S1E, Supporting Information). These results indicated consistently that SHPC reduced ROS levels, cardiomyocyte apoptosis, and pyroptosis after H/R injury in vitro.

We further investigated whether SHPC attenuated MI/R injury by communicating with other cells. We revealed a sharp increase in neutrophils during ischemia‐reperfusion by analyzing the single‐cell RNA sequencing (scRNA‐seq) data of sham and I/R heart tissues from the GSE146285 dataset (Figure 2E,F; Figure S1F, Supporting Information). Additionally, a significant increase was observed from nothing in communication between neutrophils and cardiomyocytes in the MI/R injury model (Figure 2G). Hence, the secretome may have mediated crosstalk between neutrophils and cardiomyocytes. Neutrophils were isolated from bone marrow to investigate the association of SHPC with neutrophil functions (Figure S1G,H, Supporting Information). We then treated neutrophils with SHPC before they were subjected to H/R injury (Figure 2H). The markers of N2 neutrophils (Arg1, CD206, and Tgfβ1) were upregulated (Figure 2I,J), whereas N1 neutrophil markers (Ccl3, Ccl5, and IL‐12α) were downregulated in SHPC‐treated neutrophils, indicating that SHPC‐induced neutrophil polarization. Flow cytometry and immunofluorescent staining further confirmed that SHPC‐induced neutrophil phenotype switching from N1 to N2 phenotype (Figure 2K,L). Moreover, myeloperoxidase (MPO) and matrix metallopeptidase 9 (MMP9), which are two inflammatory factors in neutrophils, significantly decreased with N1 to N2 phenotypic switching (Figure 2M). We investigated whether SHPC‐induced neutrophil phenotype polarization alleviated inflammatory damage. SHPC‐induced polarized neutrophils were co‐cultured with cardiomyocytes before undergoing H/R injury (Figure 2N). Western blot revealed that the H/R‐induced cardiomyocyte apoptosis and pyroptosis were partially attenuated when the cardiomyocytes were co‐cultured with SHPC‐induced polarized neutrophils (Figure 2O), indicating that polarized neutrophils reduce the H/R‐induced cardiomyocytes apoptosis and pyroptosis. Altogether, the above results indicated that SHPC attenuated I/R injury by reducing cardiomyocyte apoptosis and pyroptosis and mediating neutrophil polarization.

### 2.3 Stanniocalcin‐1 (Stc1) was the Key Factor in SHPC that Mediated Cardio‐Protection

**Figure 3:**
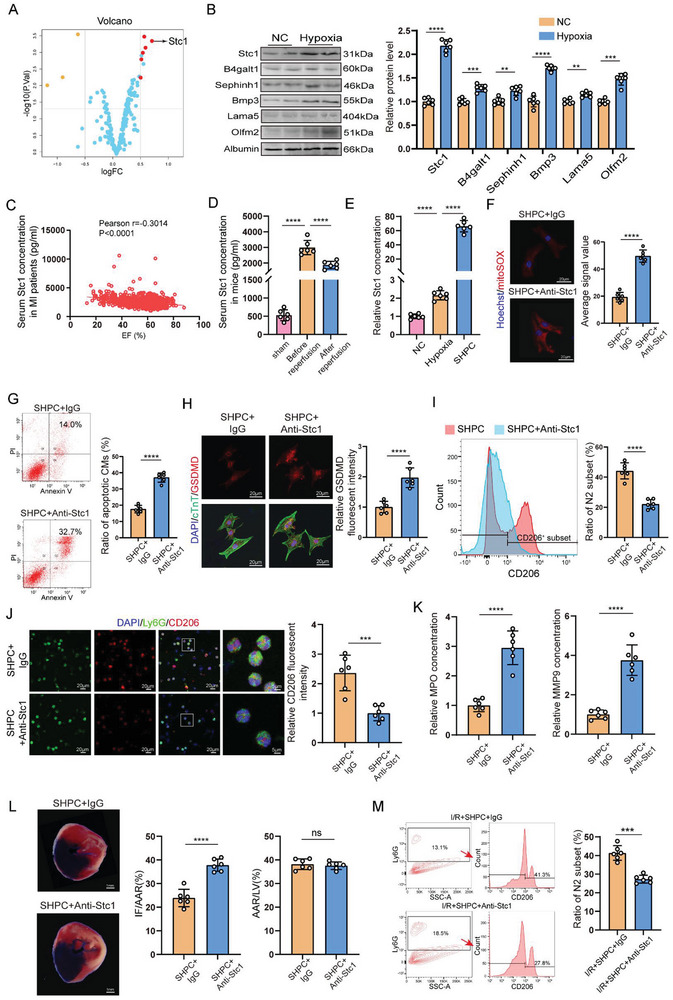
Stanniocalcin‐1 (Stc1) was the key factor in SHPC that mediated hypoxic microenvironment. A) Volcano map of differentially expressed proteins in NC and hypoxic cardiomyocyte supernatant. B) Stc1, B4galt1, Sephinh1, Bmp3, Lama5, and Olfm2 protein levels in NC and hypoxic cardiomyocyte supernatant (n = 6 cell samples). C) Correlation between serum Stc1 levels and left ventricular ejection fraction in the patients with myocardial infarction. D) ELISA assay results showing Stc1 levels in sham mice and I/R mice before and after reperfusion (n = 6 mice). E) ELISA assay results indicating Stc1 levels in cardiomyocyte supernatant after hypoxia or H/R treatment and SHPC (n = 6 supernatant samples). F) Mitochondria‐derived ROS staining in SHPC+IgG‐ or SHPC+Anti‐Stc1‐treated cardiomyocytes after H/R treatment (162 cardiomyocytes from 6 mice in the SHPC+IgG group and 173 cardiomyocytes from 6 mice in the SHPC+Anti‐Stc1 group; scale bar = 20 µm). G) Flow cytometry analysis of SHPC+IgG‐ or SHPC+Anti‐Stc1‐treated cardiomyocyte apoptosis after H/R treatment. Apoptotic cells include Annexin V(+)/PI(−) and Annexin V(+)/PI(+) cells (n = 6 cell samples). H) Immunofluorescent staining for GSDMD (red) and cTnT (green) in SHPC+IgG‐ or SHPC+Anti‐Stc1‐treated cardiomyocytes after H/R treatment (306 cardiomyocytes from 6 mice in the SHPC+IgG group and 314 cardiomyocytes from 6 mice in the SHPC+Anti‐Stc1 group; scale bar = 20 µm). I) SHPC+IgG‐ or SHPC+Anti‐Stc1‐treated neutrophils collected for flow cytometry of CD206 after H/R treatment (n = 6 cell samples). J) SHPC+IgG‐ or SHPC+Anti‐Stc1‐treated neutrophils collected for immunofluorescent staining for Ly6G (green) and CD206 (red) after H/R treatment (456 neutrophils from 6 mice in the SHPC+IgG group and 423 neutrophils from 6 mice in the SHPC+Anti‐Stc1 group; scale bar = 20 µm [three on the left] and 5 µm [rightmost]). K) ELISA results revealing MPO and MMP9 concentrations in SHPC+IgG‐ or SHPC+Anti‐Stc1‐treated neutrophil supernatants after H/R treatment (n = 6 supernatant samples). L) Representative Evans blue and TTC‐stained heart sections, and quantitative data from mice with SHPC+IgG or SHPC+anti‐Stc1 injection 24 h after I/R surgery. IF: infarct area, AAR: area at risk, LV: left ventricle (scale bar = 1 mm; n = 6 mice). M) Flow cytometry analysis of neutrophil polarization in I/R models after SHPC+IgG or SHPC+anti‐Stc1 intervention (n = 6 mice). Statistical significance was calculated with an unpaired *t*‐test in (B), (F–M), one‐way ANOVA in (D–E), and Pearson correlation in C. **P < 0.01, ***P < 0.001, and ****P < 0.0001.

To characterize the contribution of key bioactive factors derived from the SHPC, we employed secretome proteomics analysis to screen SHPC protein components. This proteomics analysis of the secretome revealed Stc1 as the major secreted protein in SHPC (**Figure** **3**A,B). We analyzed serum samples from patients with myocardial infarction (the Data are available at https://www.ahajournals.org/doi/suppl/10.1161/CIRCULATIONAHA.119.045158) and found that serum Stc1 concentrations were negatively correlated with cardiac function in these patients, indicating that hypoxic stress induced the Stc1 secretion (Table S4, Supporting Information; Figure 3C). Besides, we observed that the slightly increased serum Stc1 level during ischemia fall back after reperfusion in mouse MI/R injury models (Figure 3D). Hypoxia increased mRNA and protein expression of Stc1 in cardiomyocytes in both in vitro and in vivo (Figure S2A–D, Supporting Information). Additionally, SHPC could greatly enrich Stc1 concentration via ultrafiltration (Figure 3E).

To determine whether Stc1 was the main functional factor in SHPC, anti‐Stc1 neutralizing antibody (98‐247 aa) was utilized to eliminate the effect of Stc1 (6‐206 aa). Treatment with the anti‐Stc1 neutralizing antibody neutralized over 95% of the Stc1 content in SHPC (Figure S2E, Supporting Information). The reduction of cardiomyocyte ROS especially mtROS exerted by SHPC was counteracted after eliminating the effect of Stc1 using anti‐Stc1 (Figure 3F; Figure S2F, Supporting Information). Besides, cardiomyocyte apoptosis and pyroptosis reduction induced by SHPC were almost completely abrogated after anti‐Stc1 neutralizing antibody treatment (Figure 3G,H; Figure S2G, Supporting Information). Moreover, neutrophil polarization induced by SHPC could be reversed in the presence of an anti‐Stc1 neutralizing antibody (Figure 3I,J). Simultaneously, MPO and MMP9 levels significantly increased in the presence of anti‐Stc1 neutralizing antibody (Figure 3K). In the co‐culture system, when treating with anti‐Stc1 neutralizing antibody to reduce SHPC‐induced polarized neutrophils, the reduction of cardiomyocyte apoptosis and pyroptosis could be partially attenuated (Figure S2H, Supporting Information). This indicated that the cardioprotection mediated by SHPC between cardiomyocytes and neutrophils is dependent on Stc1.

To investigate whether Stc1 was the predominant bioactive factor in vivo, mouse MI/R injury models were myocardially injected in situ with SHPC containing neutralizing antibodies or IgG. The decrease of infarct size in the area at risk at 24 h post‐I/R by SHPC was reversed by adding an anti‐SC anti‐Stc1 neutralizing antibody (Figure 3L). Further, the reduction of cardiac injury biomarkers of cTnT, NTpro‐BNP, CK‐MB, and LDH in the plasma by SHPC was reversed by adding an anti‐Stc1 neutralizing antibody (Figure S2I–L, Supporting Information). Moreover, the decrease of HW/BW ratio and HW/TL ratio by SHPC was abrogated when intramyocardially injected with anti‐Stc1 neutralizing antibody (Figure S2M,N, Supporting Information). SHPC mediated neutrophil phenotypic switching almost counteracted when intramyocardially injected with an anti‐Stc1 neutralizing antibody (Figure 3M).

**Figure 4:**
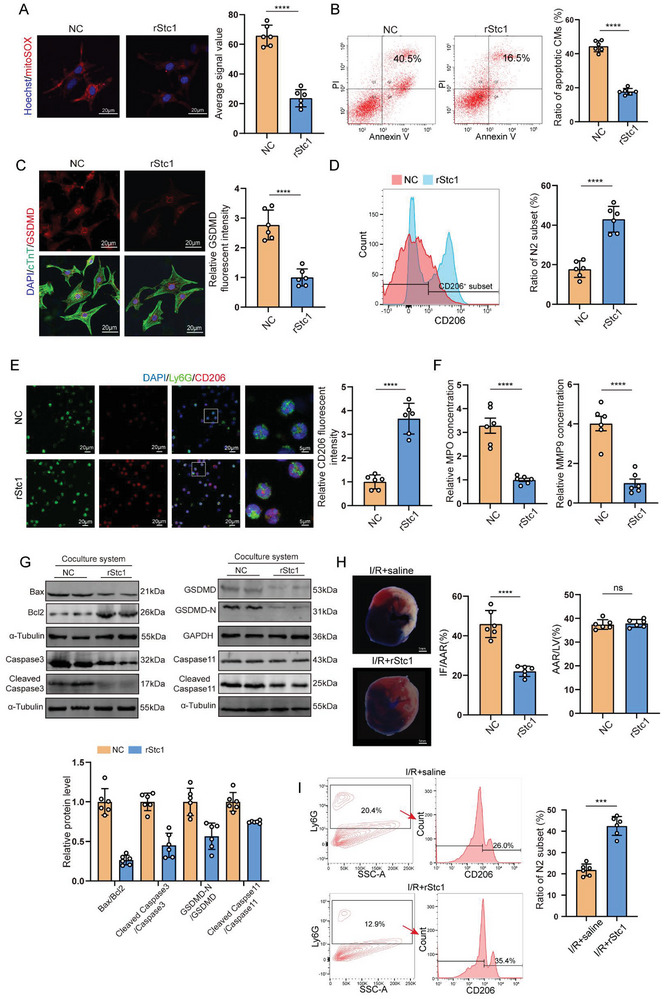
Recombinant Stc1 significantly attenuated MI/R injury by reducing cardiomyocyte apoptosis and pyroptosis and promoting neutrophil polarization. A) Mitochondria‐derived ROS staining in NC‐ or rStc1‐treated cardiomyocytes after H/R treatment (148 cardiomyocytes from 6 mice in the NC group and 127 cardiomyocytes from 6 mice in the rStc1 group; scale bar = 20 µm). B) Flow cytometry analysis of NC‐ or rStc1‐treated cardiomyocyte apoptosis after H/R treatment. Apoptotic cells include Annexin V(+)/PI(−) and Annexin V(+)/PI(+) cells (n = 6 cell samples). C) Immunofluorescent staining for GSDMD (red) and cTnT (green) in NC‐ or rStc1‐treated cardiomyocytes after H/R treatment (322 cardiomyocytes from 6 mice in the NC group and 354 cardiomyocytes from 6 mice in the rStc1 group). D) NC‐ or rStc1‐treated neutrophils collected for flow cytometry of CD206 after H/R treatment (n = 6 cell samples). E) NC‐ or rStc1‐treated neutrophils collected for immunofluorescent staining for Ly6G (green) and CD206 (red) after H/R treatment (428 neutrophils from 6 mice in the NC group and 449 neutrophils from 6 mice in the rStc1 group; scale bar = 20 µm [three on the left] and 5 µm [rightmost]). F) ELISA results demonstrating MPO and MMP9 concentrations in NC‐ or rStc1‐treated neutrophil supernatants after H/R treatment (n = 6 supernatant samples). G) Bax, Bcl2, Cleaved caspase 3, Caspase 3, Cleaved GSDMD‐N, GSDMD, Cleaved caspase 11, and Caspase11 protein levels in cardiomyocytes co‐cultured with NC‐ or rStc1‐treated neutrophils (n = 6 cell samples). H) Representative Evans blue and TTC‐stained heart sections, and quantitative data from mice with saline or rStc1 injection 24 h after I/R surgery. IF: infarct area, AAR: area at risk, LV: left ventricle (scale bar = 1 mm; n = 6 mice). I) Flow cytometry analysis of neutrophil polarization in I/R+saline‐ and I/R+rStc1‐treated mice (n = 6 mice). Statistical significance was calculated with an unpaired *t*‐test in (A–I); ***P < 0.001 and ****P < 0.0001.

To further determine whether Stc1 could be an effective substitute for SHPC, rStc1 was utilized to mimic the role of SHPC. Titration ELISA showed that Stc1 has a good affinity with receptors in both cardiomyocytes and neutrophils by determinating dissociation constant (Kd) (Kd in cardiomyocytes: 2.1875·10^−9^ mol L^−1^; Kd in neutrophils:1.25·10^−9^ mol L^−1^) (Figure S3A,B, Supporting Information). We determined the optimal concentration of rStc1 intervention at the point when the receptors get saturated. The titration ELISA demonstrated 100 and 60 ng ml^−1^ as the optimal concentrations (the concentration of rStc1 when the concentration of the complex is closest to the peak) of rStc1 for cardiomyocytes and neutrophils, respectively (Figure S3A,B, Supporting Information). Treatment with rStc1 significantly reduced cardiomyocyte ROS (**Figure** **4**A; Figure S3C, Supporting Information). Additionally, the H/R‐induced cardiomyocyte apoptosis and pyroptosis could be reduced by rStc1 (Figure 4B,C; Figure S3D, Supporting Information). In vitro experiments confirmed that neutrophils underwent N1‐N2 phenotypic switching using rStc1 intervention (Figure 4D–F). Neutrophils treated with rStc1 caused significantly less cardiomyocyte apoptosis and pyroptosis in the co‐culture system (Figure 4G). In vivo, mice induced with MI/R injury were intramyocardially injected with the recombinant protein before reperfusion. Analysis revealed a global reduction in biomarker levels of cardiac injury in mice injected with rStc1 after MI/R injury (Figure S3E–H, Supporting Information). Intramyocardial rStc1 injection strikingly decreased the injured size at 24 h post‐I/R as compared with untreated MI/R mice (Figure 4H). Furthermore, the HW/BW and HW/TL ratios significantly decreased in the rStc1 group at 4 weeks after I/R surgery as compared with the control group (Figure S3I,J, Supporting Information). Additionally, rStc1 treatment inhibited inflammatory infiltration and induced neutrophil polarization in mice that underwent MI/R injury, as measured by flow cytometry (Figure 4I).

Overall, these data indicated Stc1 as the main bioactive factor of SHPC that orchestrated cardiomyocyte self‐protective mechanisms and modulated neutrophil polarization.

### 2.4 Stc1 Mediated Cardio‐Protection via the Calcium‐Sensing Receptor (CaSR)

**Figure 5:**
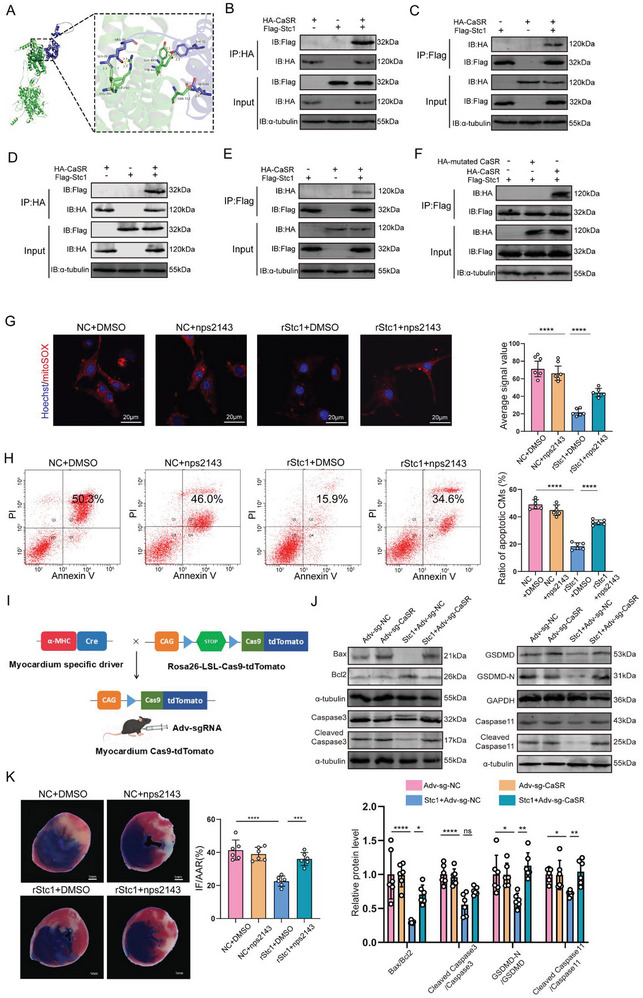
Stc1‐mediated cardioprotective effects are mediated through the CaSR. A) Molecular docking of Stc1 (green) and CaSR (purple). B–E) Stc1‐CaSR Co‐immunoprecipitation assays utilizing cardiomyocytes transfected with HA‐CaSR and Flag‐Stc1 constructs individually or together. F) Stc1‐CaSR Co‐immunoprecipitation assays utilizing cardiomyocytes transfected with HA‐mutated CaSR, HA‐CaSR, and Flag‐Stc1 constructs individually or together. G) Mitochondria‐derived ROS staining in the NC+DMSO, NC+nps2143, rStc1+DMSO, or rStc1+nps2143 groups after H/R treatment (including 155, 172, 166, and 148 cardiomyocytes from 6 mice in each group, respectively; scale bar = 20 µm). H) Flow cytometry analysis of NC+DMSO, NC+nps2143, rStc1+DMSO, or rStc1+nps2143 groups after H/R treatment. Apoptotic cells include Annexin V(+)/PI(−) and Annexin V(+)/PI(+) cells (n = 6 cell samples). I) Scheme showing the process of obtaining mice myocardially expressing Cas9, followed by the delivery of Adv‐expressing sgRNA. (J) Bax, Bcl2, Cleaved caspase 3, Caspase 3, Cleaved GSDMD‐N, GSDMD, Cleaved caspase 11 and Caspase11 protein levels in I/R mice myocardially expressing Cas9 (n = 6 mice). K) Representative Evans blue and TTC‐stained heart sections, and quantitative data from mice 24 h after I/R surgery. IF: infarct area, AAR: area at risk, LV: left ventricle (scale bar = 1 mm; n = 6 mice). Statistical significance was calculated with a one‐way ANOVA in G‐H, J‐K; **P < 0.01, ***P < 0.001, and ****P < 0.0001.

We explored the mechanism by which Stc1 mediated endogenous cardio‐protection. Then, protein–protein interaction (PPI) network analysis was conducted to predict the receptors that interacted with Stc1. The PPI analysis revealed CaSR as a potential Stc1 receptor with a high score (Figure S4A, Supporting Information), and molecular docking, Co‐immunoprecipitation assays, and site mutation experiment indicated a strong direct interaction between stc1 and CaSR (**Figure** **5**A–F; Figure S4B, Supporting Information). Additionally, the reduction in cardiomyocyte ROS, apoptosis, and pyroptosis was almost abrogated when CaSR was blocked by nps2143, which is a CaSR inhibitor (Figure 5G,H; Figure S4C,D, Supporting Information). CaSR was expressed in neutrophils, thus we investigated whether Stc1 promoted neutrophil polarization via CaSR. Immunofluorescent staining revealed that nps2143 largely counteracted the role of Stc1 in promoting the neutrophil phenotypic switch (Figure S4E,F, Supporting Information). Additionally, MPO and MMP9 level reduction exerted by rStc1 was reversed after nps2143 blocked the CaSR (Figure S4G, Supporting Information).

We established cardiomyocyte‐specific CaSR knockout mice to identify whether Stc1 mediated cardio‐protection via the CaSR (Figure 5I). The transduction efficiency of Adv‐sgRNA in the heart tissue was ≈80% (Figure S4H, Supporting Information). RT‐qPCR and Western blotting confirmed the efficient deletion of CaSR expression in Cas9‐tdTomato mouse hearts (Figure S4I,J, Supporting Information). The Stc1‐mediated reduction of cardiomyocyte apoptosis and pyroptosis was reversed in the cardiomyocyte‐specific CaSR knockout mice (Figure 5J). Moreover, the infarct size decrease in the area at risk at 24 h post‐I/R by rStc1 was reversed by adding nps2143 (Figure 5K)

Collectively, these results indicate that cardiomyocytes secreted Stc1 into the medium reduced cardiomyocyte apoptosis/pyroptosis and communicated with neutrophils to trigger polarization via CaSR.

### 2.5 Stc1 Mediated Cardioprotective Effects through the CaSR/NOS2/Stat3 (Ser727) axis

**Figure 6:**
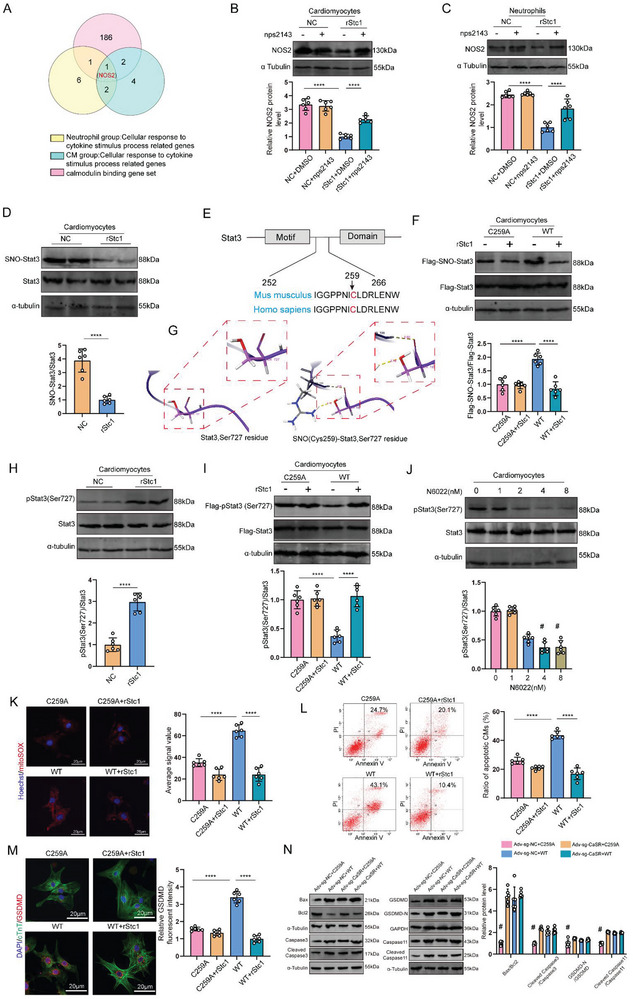
Stc1‐mediated cardioprotective effects through the CaSR/NOS2/Stat3 (Ser727) axis. A) A Venn diagram illustrating the genes shared between cellular response to cytokine stimulus pathways in cardiomyocytes and neutrophils, and the calmodulin‐binding gene set. B,C) NOS2 protein levels in the NC+DMSO, NC+nps2143, rStc1+DMSO, or rStc1+nps2143 group cardiomyocytes and neutrophils after H/R treatment (n = 6 cell samples). D) SNO‐Stat3 protein levels in the NC and rStc1 group cardiomyocytes after H/R treatment (n = 6 cell samples). E) A comparison of the protein structure and sequence homology analysis for amino acids 252–266 in human and mouse Stat3. F) SNO‐Stat3 protein levels in cardiomyocytes transfected with Flag‐tagged wild‐type Stat3 (WT) or its C259A mutant and treated with rStc1 after H/R intervention (n = 6 cell samples). G) Structural modeling of Stat3 at Ser727 residue before and after S‐nitrosylation at Cys259 (dashed line representing hydrogen bonding). H) pStat3 (Ser727) protein levels in cardiomyocytes treated with rStc1 after H/R intervention (n = 6 cell samples). I) pStat3 (Ser727) protein levels in cardiomyocytes transfected with Flag‐tagged wild‐type Stat3 (WT) or its C259A mutant and treated with rStc1 after H/R intervention (n = 6 cell samples). J) pStat3 (Ser727) protein levels in cardiomyocytes after N6022 treatment, a specific inhibitor of S‐nitrosylation (n = 6 cell samples). K) Mitochondria‐derived ROS staining in C259A, C259A+rStc1, WT, or WT+rStc1 group after H/R treatment (including 143, 152, 138, and 162 cardiomyocytes from 6 mice in each group, respectively; scale bar = 20 µm). L) Flow cytometry analysis of the C259A, C259A+rStc1, WT, or WT+rStc1 group after H/R treatment. Apoptotic cells include Annexin V(+)/PI(−) and Annexin V(+)/PI(+) cells (n = 6 cell samples). M) Immunofluorescent staining for GSDMD (red) and cTnT (green) in the C259A, C259A+rStc1, WT, or WT+rStc1 group after H/R treatment (including 155, 148, 144, and 132 cardiomyocytes from 6 mice in each group, respectively; scale bar = 20 µm). N) Bax, Bcl2, Cleaved caspase 3, Caspase 3, Cleaved GSDMD‐N, GSDMD, Cleaved caspase 11 and Caspase11 protein levels in I/R mice myocardially expressing Cas9 and injected with rStc1 (n = 6 mice). Statistical significance was calculated with an unpaired *t*‐test in D and H, a one‐way ANOVA in (B,C,F,I–N); ****P < 0.0001, # *P* < 0.05 compared with other groups.

To identify the exact signaling targets activated by Stc1 binding to CaSR, we performed RNA sequencing (RNA‐seq) of cardiomyocytes and neutrophils after Stc1 intervention to identify the exact signaling targets activated by Stc1 binding to CaSR. RNA‐seq transcriptome analysis determined 779 and 639 genes in cardiomyocytes and 712 and 661 in neutrophils that were significantly upregulated and downregulated, respectively (Figure S5A,B, Supporting Information). The differentially expressed genes were involved in the cellular response to cytokine stimulus in neutrophils and cardiomyocytes, as revealed by Gene Ontology enrichment analysis (Figure S5C,D, Supporting Information). Of the differentially expressed genes involved in the cellular response to cytokine stimulus progress (Figure S5E,F, Supporting Information), NOS2 was the only overlapping differentially expressed gene in the cardiomyocyte, neutrophil, and calmodulin‐binding gene sets (**Figure** **6**A). Additionally, molecular rescue experiments conducted in neutrophils and cardiomyocytes confirmed NOS2 as a signaling target for CaSR activated by Stc1 (Figure 6B,C).

NOS2‐mediated protein S‐nitrosylation is an essential mediator of nitric oxide‐dependent cardiac homeostasis, and Stat3 protein is a key player mediating this cardioprotective effect of IPC.^[^ ^14^ ^]^ Our RNA‐seq data revealed the involvement of Stc1 in the JAK‐Stat pathway and nitric oxide metabolic process (Figure S5C,D, Supporting Information). The NO level, a key mediator for NOS2‐mediated S‐nitrosylation, was reduced in cardiomyocytes and neutrophils after Stc1 intervention (Figure S6A,B, Supporting Information). We speculated the involvement of NOS2‐mediated S‐nitrosylation of Stat3 (SNO‐Stat3) in mediating the cardioprotective effect of Stc1. Western blotting revealed that Stc1 mediated Stat3 denitrosylation in cardiomyocytes (Figure 6D). Bioinformatics (GPS‐SNO 1.0) analysis determined the Stat3‐Cys259 position as a potential S‐nitrosylation residue, and this site was conserved in human and mouse Stat3 amino acid sequences (Figure 6E). We constructed wild‐type Flag‐tagged Stat3 (WT) and its denitrosylation variant with a mutation at the consensus cysteine residue (Cys259Ala; termed the C259A) to confirm the Cys259 position as the target of Stc1‐mediated SNO‐Stat3. We revealed that the SNO‐Stat3 level decreased when the Cys259 site was mutated (Figure 6F), indicating the Cys259 site as the target Cys residue of Stc1‐mediated SNO‐Stat3.

Stat3 phosphorylation is an important form of its activation, potentially linked to SNO‐Stat3. Molecular dynamics simulations revealed that Ser727 was exposed to wild‐type Stat3 and could be easily phosphorylated. When Cys259 was S‐nitrosylated, the exposed Ser727 residue turned inside and formed two hydrogen bond interactions (2.05 Å and 2.16 Å) with the Arg729 site that prevented Ser727 phosphorylation, as observed in a 20‐ns molecular dynamics simulation (Figure 6G; Figure S6C, Supporting Information). Western blot analysis revealed that rStc1 augmented the p‐Ser727‐Stat3 protein level in cardiomyocytes (Figure 6H). Flag‐p‐Ser727‐Stat3 levels increased in cardiomyocytes when the C259 site was mutated (Figure 6I), indicating Ser727 as the phosphorylation site of Stat3. The pStat3 levels gradually decreased as the concentration increased when the cardiomyocytes were treated with different concentrations of N6022, a specific inhibitor of S‐nitroso glutathione reductase (Figure 6J). The rescue experiment confirmed SNO‐Stat3 as a key mechanism by which Stc1 regulated mtROS level, apoptosis, and pyroptosis (Figure 6K–M). Collectively, these data indicated that Stc1‐mediated S‐nitrosylation influenced cardiomyocyte apoptosis and pyroptosis through the phosphorylation of Stat3 at the Ser727 site.

Similarly, we investigated the effect of NOS2‐mediated S‐nitrosylation on the Stat3 phosphorylation at Ser727 in neutrophils. Western blotting revealed that Stc1 mediated Stat3 denitrosylation in neutrophils (Figure S6D, Supporting Information). We revealed that the SNO‐Stat3 level decreased when the Cys259 site was mutated (Figure S6E, Supporting Information), and rStc1 augmented the p‐Ser727‐Stat3 protein level in neutrophils (Figure S6F, Supporting Information). Flag‐p‐Ser727‐Stat3 levels increased in neutrophils when the C259 site was mutated (Figure S6G, Supporting Information), indicating Ser727 as the phosphorylation site of Stat3. The pStat3 levels gradually decreased as the concentration increased when the neutrophils were treated with different concentrations of N6022, a specific S‐nitroso glutathione reductase inhibitor (Figure S6H, Supporting Information). The rescue experiment revealed that NOS2‐mediated S‐nitrosylation regulated neutrophil phenotype switching through Stat3 phosphorylation at Ser727 (Figure S6I,J, Supporting Information). The MPO and MMP9 levels decreased by rStc1 were reversed when the Cys259 site was mutated (Figure S6K, Supporting Information). In vivo experiments further confirmed that Cys259 regulated the rStc1 cardioprotective effect (Figure 6N). Altogether, these results indicate that Stc1 bonded to CaSR, thereby activating the CaSR/NOS2/Stat3 (Ser727) axis to mediate cardioprotective effects.

### 2.6 Pericardial Delivery of rStc1‐Encapsulated Hydrogel Improved Long‐Term Cardiac Function

**Figure 7:**
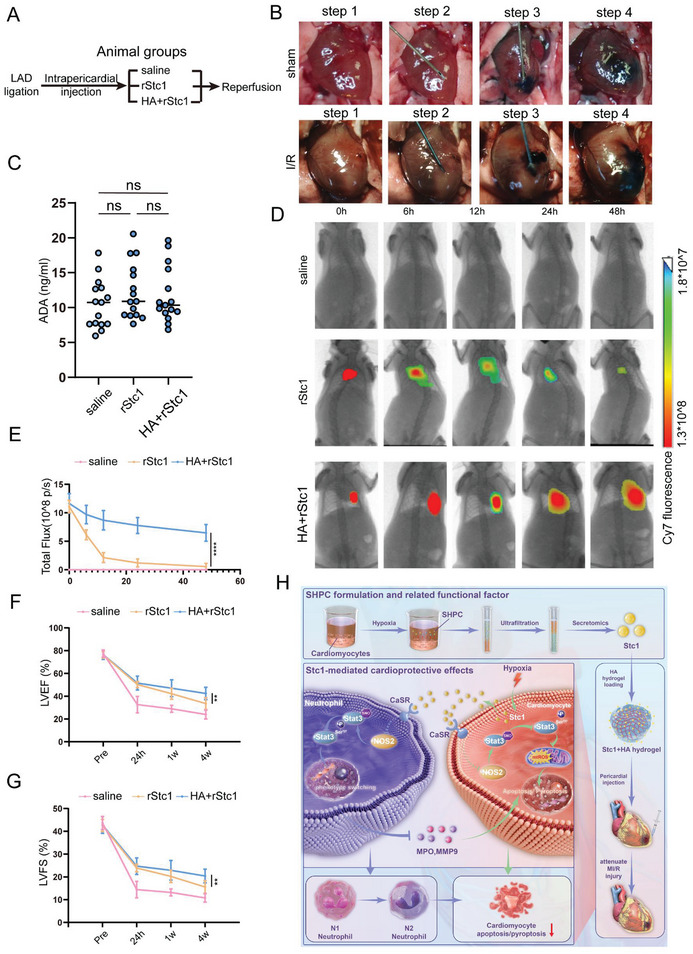
Pericardial injection of rStc1‐encapsulated hydrogel for long‐term cardiac injury repair. A) Treatment groups. B) Images showing the intrapericardial injection process in myocardial ischemia‐reperfusion mice. C) ADA detection in MI/R mice injected with saline, rStc1, and HA+rStc1 (n = 15 mice). D) Live imaging was carried out to detect rStc1 biodistribution. E) Quantitative analysis of rStc1 retention in the heart (n = 3 mice). F,G) Cardiac function was analyzed using echocardiography at pre‐I/R surgery, 24 hours, 1 week, and 4 weeks post‐I/R surgery (n = 6 mice). LVEF, left ventricular ejection fraction; LVFS, left ventricular fraction shortening. H) A schematic illustration showing the hypoxia microenvironment preconditioning mimicked by SHPC attenuated MI/R injury via Stc1‐mediated cardiomyocyte self‐protection and neutrophil polarization. Statistical significance was calculated using two‐way ANOVA in (D–G); **P < 0.01, and ****P < 0.0001.

We developed a hydrogel formulation encapsulating recombinant Stc1 protein (rStc1‐encapsulated hydrogel) for intrapericardial injection to evaluate the effectiveness and safety of the pericardial delivery of rStc1 (**Figure** **7**A,B). We conducted ADA detection to evaluate the immunogenicity of rStc1 and revealed that rStc1 did not cause significant immune rejection (Figure 7C). Using intravital imaging, we revealed that pericardial delivery of the rStc1‐encapsulated hydrogel extended the therapeutic time window of rStc1 by at least 48 h (Figure 7D,E). Additionally, echocardiography revealed that the pericardial delivery of the rStc1‐encapsulated hydrogel extended the therapeutic time window of rStc1 and improved long‐term cardiac function (Figure 7F,G). In brief, pericardial injection of rStc1 hydrogel is an effective approach to prevent and treat cardiac I/R injury.

## 3 Discussion

To facilitate the clinical translation of ischemic preconditioning (IPC) therapy, this study innovatively applied an ultrafiltered SHPC to mimic the microenvironment of IPC. This approach successfully attenuated myocardial ischemia/reperfusion (MI/R) injury by reducing cardiomyocyte apoptosis, pyroptosis, and ROS. We then determined Stc1 as the key functional factor in SHPC that exhibits cardio‐protection. Mechanistically (Figure 7H), hypoxic‐preconditioned cardiomyocytes secreted Stc1 into the microenvironment and bound to CaSR decreasing the NOS2 level, thereby promoting the S‐nitrosylation of Stat3 phosphorylation at Ser727, which ultimately reduced cardiomyocyte apoptosis/pyroptosis in an autocrine mechanism. Stc1 simultaneously mediated cardiomyocyte‐neutrophil crosstalk that triggered neutrophil polarization to reduce inflammatory damage via the above CaSR/NOS2/Stat3 axis in a paracrine mechanism. Notably, we determined a novel site of S‐nitrosylation in STAT3 phosphorylation at Ser727, and targeting this site could exert the double effect of mtROS elimination and neutrophil polarization modulation, thereby providing evidence that this novel site is a valuable target for alleviating I/R injury. Another important novel finding was that we designed a hydrogel‐encapsulated rStcl to facilitate the functional factor Stc1 translation into clinical. Our work has great translational potential to the clinic for attenuating MI/R injury in the future.

### 3.1 SHPC is a Clinically Feasible Alternative to IPC for Attenuating MI/R Injury

This study harnessed the cardiomyocyte secretome, which interacts directly with membrane receptors to perform its functions through autocrine and paracrine mechanisms within the microenvironment. Moreover, this study innovatively applied ultrafiltration to acquire the SHPC that enabled small molecule accumulation in concentrated solutions, thereby facilitating the investigation of active components within the secretome. Recently, interest in cell secretome‐based therapy as an alternative therapeutic option increased due to the possible side effects of cell‐based therapy. Cell secretome‐based therapy appeared as a promising therapeutic approach since the secretome is less immunogenic but exerts similar biological actions as compared to cell‐based therapy. The secretome possesses multifunctional properties that orchestrate cell proliferation and growth, influence cell differentiation, regulate immune responses, and facilitate critical signal transmission.^[^ ^15^ ^]^ Additionally, the secretome, such as conditioned media or exosomes, provides many advantages for clinical translation of therapy, including a wide range of acquisition sources, easy storage, and high biological safety.^[^ ^16^ ^]^ Based on these advantages of secretome‐based therapy, this study applying the secretome derived from hypoxia‐preconditioned cardiomyocytes to attenuate MI/R injury showed great potential in facilitating the clinical translation of IPC therapy.

### 3.2 The Cardioprotective Effect of SHPC was Attributable to Neutrophil Polarization and ROS Reduction

Reactive oxygen species (ROS) generation and neutrophil activation are generally considered the principal mechanisms of ischemia/reperfusion (I/R) injury.^[^ ^17^ ^]^ The pivotal role of ROS and neutrophils at the early inflammatory response stage of MI/R makes them potential therapeutic targets for ameliorating MI/R injury. Our study demonstrated that SHPC promoted the neutrophils switched from proinflammatory N1 phenotype into anti‐inflammatory N2 phenotype, which further reduced the release of MPO and MMP9, to suppress the inflammatory response. Previous studies have revealed that eliminating circulating neutrophils using anti‐neutrophil antibodies, inhibiting adhesion molecules, or depleting the chemokine complement mitigates MI/R injury. However, neutrophil elimination may disturb homeostasis. Increasing evidence indicated that neutrophils can be subdivided into multiple functional subgroups, among which N1 neutrophils are proinflammatory neutrophils and N2 neutrophils are anti‐inflammatory neutrophils. Triggering neutrophil polarization in response to a hypoxic microenvironment is a more promising approach. Our study revealed that SHPC or rStc1 treatment promoted the neutrophils’ switching from proinflammatory N1 phenotype into anti‐inflammatory N2 phenotype that further reduced the release of MPO and MMP9 to suppress the inflammatory response. Our strategy focuses on triggering neutrophil polarization rather than simply eliminating circulating neutrophils to avoid the adverse effects of disturbing homeostasis.^[^ ^18^, ^19^ ^]^ Moreover, our study demonstrated that SHPC or rStc1 treatment reduced the ROS especially the mtROS to ameliorate MI/R injury. Therefore, SHPC is an effective and safe strategy to attenuate MI/R injury by collaboratively regulating mtROS and neutrophil polarization.

### 3.3 Stc1 as an Effective Substitute for SHPC Facilitates Clinical Translation

Identifying key bioactive factors in the SHPC is crucial for the clinical translation of this approach. This study determinated Stc1 as an effective substitute for SHPC to attenuate MI/R injury. Previous studies have revealed that mammalian Stc1 is associated with diverse cellular processes, including oxidative stress, inflammation, cell death, and impaired calcium homeostasis.^[^ ^20^, ^21^ ^]^ Reportedly, the expression of Stc1 increased in the culprit coronary plaques of patients with acute myocardial infarction (AMI) as compared with stable angina.^[^ ^22^ ^]^ Interestingly, our results revealed that serum Stc1 concentration was negatively correlated with cardiac function in patients with AMI, indicating the important role of Stc1 in AMI.^[^ ^23^ ^]^ A recent study showed that recombinant human Stc1 injection alleviated MI/R injury by inhibiting inflammation and apoptosis.^[^ ^24^, ^25^, ^26^ ^]^ These multiple lines of evidence confirm the view that Stc1 plays a crucial role in MI/R injury. However, previous studies ignored the mechanism of Stc1 secretion and its exact roles in the hypoxia microenvironment during I/R injury. This study is the first to reveal that Stc1 was secreted by hypoxic cardiomyocytes, and hypoxic preconditioning improved Stc1 production to reduce cardiomyocyte apoptosis and pyroptosis in an autocrine mechanism. Stc1 simultaneously mediated cardiomyocyte‐neutrophil crosstalk in the hypoxia microenvironment that triggered neutrophil polarization to reduce inflammatory damage in a paracrine mechanism.

### 3.4 Molecular Mechanism Underlying Stc1‐Mediated Cardioprotective Effects

Our study further revealed that Stc1 exerted dual cardioprotective effects via the CaSR/NOS2/Stat3 pathway. Previous studies have indicated that Stc1 alleviated MI/R injury, but the underlying mechanisms remain largely unknown. Stc1 was originally determined as a secreted Ca2+‐regulating hormone in bony fishes. The CaSR, which is a member of the class C of G‐proteins coupled receptors, plays a pivotal role in calcium homeostasis and cardio‐protection.^[^ ^27^ ^]^ This study revealed that the Stc1 directly interacted with CaSR in the hypoxic microenvironment via PPI network analysis and Co‐immunoprecipitation assays. Importantly, our in vitro and in vivo study further confirmed that CaSR regulated the Stc1 mediated cardioprotective effect with the CaSR inhibitor. CaSR is known to activate the phosphatidylinositol calcium messenger system and regulate calmodulin which further modulates NOS2 expression.^[^ ^28^, ^29^ ^]^ By applying RNA‐seq analysis, we revealed NOS2 as a signaling target for CaSR activated by Stc1. Our molecular rescue experiments confirmed that Stc1‐ligated CaSR activated NOS2. NOS2 primarily mediates S‐nitrosylation, a redox‐sensitive post‐translational modification.^[^ ^30^, ^31^ ^]^ S‐nitrosylation, as a covalent post‐translational modification, has been confirmed to affect cardiovascular system homeostasis by regulating enzyme activity, protein–protein interactions, or signal transduction of target proteins. Previous studies have indicated NOS2‐mediated protein S‐nitrosylation as an essential mediator of nitric oxide‐dependent cardiac homeostasis, whereas Stat3 protein activation is recognized as a RISK‐free pathway that confers protection against IPC.^[^ ^14^ ^]^ Our research revealed that Stc1 that directly binds to CaSR decreased the NOS2‐mediated S‐nitrosylation level, thereby promoting Stat3 phosphorylation at Ser727 that activated the JAK‐Stat3 pathway to confer cardio‐protection. Notably, we determined Ser727, regulated by S‐nitrosylation, as a novel phosphorylation site of Stat3.

The phosphorylation modification sites of Tyr705 and Ser727 are considered the most important phosphorylation modification of Stat3, but only Tyr705 phosphorylation of Stat3 mediated by S‐nitrosylation has been reported. Additionally, phosphorylation modification at Ser727 is closely associated with mtROS production. This study revealed, for the first time, that Stat3 phosphorylation at Ser727 exerted a double effect of mtROS elimination and neutrophil polarization modulation, indicating that S‐nitrosylation‐mediated Stat3 phosphorylation at Ser727 may be a valuable target for alleviating I/R injury.

### 3.5 Pericardial Injection of rStc1‐Encapsulated Hydrogels Helps the Clinical Translation of rStc1

The novel finding that rStc1 mimics the role of SHPC in attenuating I/R injury promotes the investigation of its application for clinical translation. In situ injection of recombinant proteins in the myocardium poses a risk of cardiac damage, and the protective window of time in the heart is short which would greatly hinder clinical translation. Intrapericardial injection is an efficient and safe cardiac‐targeted delivery system for polymerized hydrogels formed in situ at the injection site. Hydrogels are popular for their capacity to extend drug retention time.^[^ ^32^ ^]^ Ke et al. have recently demonstrated a novel drug delivery method that involves intrapericardial injection (iPC) of an exosome‐loaded hydrogel, demonstrating the pericardial cavity as an ideal natural mold for injectable hydrogels, which form a uniform cardiac patch that covers the entire heart.^[^ ^13^ ^]^ In this study, we developed a hydrogel formulation that encapsulates the rStc1 protein and demonstrated that the pericardial delivery of rStc1‐encapsulated hydrogel extended the therapeutic time window of rStc1 and improved long‐term cardiac function. Pericardial rStc1‐encapsulated hydrogel injection is an effective method for attenuating MI/R injury. Surgical open‐chest operation for treating MI/R injury is not a feasible option in the clinic; thus, intrapericardial injection with the help of an endoscope may achieve minimally invasive delivery. Future translational research into the safety and feasibility of pericardial injection of rStc1‐encapsulated hydrogel with endoscopic assistance is warranted.

### 3.6 Limitation

This study has several limitations. First, we primarily focused on the effect of SHPC on cardiomyocytes. Angiogenesis also plays a crucial role in cardiac repair post‐I/R injury; thus, the association of SHPC with angiogenesis in the heart warrants further investigation. Second, this study mainly examined the Stc1 as the key secreted factor in SHPC; hence, whether other key secreted factors exerted a cardiac protective effect similar to SHPC required further research. Third, Stc1 played a key role in regulating the balance of Ca2^+^ and phosphate metabolism in many cell types, and this study mainly focuses on its role in the neutrophils and cardiomyocytes; thus, the association of Stc1 with other cell types remains unclear. Lastly, we did not demonstrate the CaSR/NOS2/Stat3 pathway through pharmacological interventions of NOS2/Stat3 pathway in the CaSR knockout mice in vivo, future experiment is needed.

## 4 Conclusion

This study revealed that IPC microenvironment, mimicked by SHPC, attenuated MI/R injury and improved cardiac function. This effect is attributed to Stc1‐mediated dual mechanisms that improved cardiomyocyte self‐protection and promoted neutrophil polarization via the CaSR/NOS2/Stat3 pathway. Pericardial delivery of a recombinant rStc1‐encapsulated hydrogel has extended the therapeutic time window of rStc1, improving long‐term cardiac function. Our results indicated that SHPC and hypoxia preconditioning factor Stc1 functioned as a clinically feasible alternative to IPC for attenuating MI/R injury.

## 5 Experimental Section

### Mice

The C57BL/6 mice used in this study were provided by the Southern Medical University in Guangzhou, China. A Cre‐dependent Cas9 knock‐in mouse model (Rosa26‐LSL‐Cas9‐tdTomato) was purchased from Shanghai Model Organisms Center, Inc. α‐MHC‐Cre mice were provided by Dr. Kunfu Ouyang from Peking University Shenzhen Graduate School, China. The animals were housed in a controlled, specific pathogen‐free environment at Southern Medical University. Mice, comprising equal numbers of males and females and aged 8–10 weeks, were randomly assigned to different groups. The researchers responsible for data collection were blinded to the treatment conditions. Ethical compliance was upheld following the principles outlined in Directive 2010/63/EU of the European Parliament. All animal procedures were approved by the Institutional Animal Care and Use Committee of Southern Medical University.

### In Vivo Myocardial I/R Protocol

The surgical protocol employed to induce MI/R closely adhered to previously published methodologies.^[^ ^12^ ^]^ C57BL/6 mice were randomly categorized into the sham and MI/R groups and were anesthetized by inhalation anesthesia using 2% isoflurane. The left anterior descending coronary artery (LAD) was ligated for 30 min, after which the slipknot was opened to allow reperfusion. Sham group mice underwent analogous surgical procedures, with the sole exception of suture passage under the left anterior descending artery without ligation. Myocardial ischemia was assessed based on ST‐segment elevation on electrocardiogram (ECG). The in vivo injection concentration of rStc1 was 5 µg kg^−1^. The nonTomy I/R models were developed with preserved pericardium for intrapericardial injection. Briefly, nonTomy I/R models were established by ligating the pericardium together with the left anterior descending artery (LAD) for 30 min, and the slipknot was then opened to enable reperfusion.^[^ ^13^ ^]^

### Ultrafiltration Solution Preparation

The ultrafiltration solution was prepared with an Amicon Ultra filter device (Millipore), following the manufacturer's instructions. Briefly, each sample was introduced into an Amicon Ultra filter device. The Amicon device was centrifuged at a maximum speed of 4000 ×g for ≈15–60 min using a swinging bucket rotor. The device was oriented with the membrane panel facing upward and centrifuged at a maximum speed of 5000 ×g for ≈15–60 min when using a fixed‐angle rotor. A pipette was inserted into the bottom of the filter device and the sample was withdrawn using a side‐to‐side sweeping motion to ensure complete recovery to retrieve the concentrated solute. The resulting ultrafiltrate was stored in a centrifuge tube.

### Statistical Analysis

All data were presented as the means ± standard deviation, and Statistical Package for the Social Sciences version 18.0 was used for result analyses. A normality test was conducted for all continuous variables. An unpaired Student's *t*‐test was utilized to analyze the two groups. One‐way analysis of variance (ANOVA) followed by Bonferroni's multiple comparisons test or two‐way ANOVA followed by Sidak's test were conducted to analyze multiple groups. The nonparametric Mann–Whitney U test was conducted to compare the two independent groups for variables with an abnormal distribution. The survival rate was determined using the Kaplan–Meier method, and differences between survival curves were identified with the log‐rank (Mantel–Cox) test. A *P*‐value of <0.05 was considered statistically significant.

More detailed descriptions of the materials and methods are presented in the Supplementary Information.

## Conflict of Interest

The authors declare no conflict of interest.

## Supporting information



Supporting Information

## Data Availability

The data that support the findings of this study are available from the corresponding author upon reasonable request.

## References

[1] G. Heusch , B. J. Gersh , Eur. Heart J. 2017, 38, 774. 27354052 10.1093/eurheartj/ehw224

[2] C. E. Murry , R. B. Jennings , K. A. Reimer , Circulation. 1986, 74, 1124. 3769170 10.1161/01.cir.74.5.1124

[3] S. Wang , H. Li , N. He , Y. Sun , S. Guo , W. Liao , Y. Liao , Y. Chen , J. Bin , Int. J. Cardiol. 2017, 227, 882. 27908607 10.1016/j.ijcard.2016.11.278

[4] R. A. Kloner , Circulation. 2009, 119, 776. 19221230 10.1161/CIRCULATIONAHA.108.832832

[5] C. M. Cao , Y. Zhang , N. Weisleder , C. Ferrante , X. Wang , F. Lv , Y. Zhang , R. Song , M. Hwang , L. Jin , J. Guo , W. Peng , G. Li , M. Nishi , H. Takeshima , J. Ma , R. P. Xiao , Circulation. 2010, 121, 2565. 20516375 10.1161/CIRCULATIONAHA.110.954628

[6] D. Shan , S. Guo , H. K. Wu , F. Lv , L. Jin , M. Zhang , P. Xie , Y. Wang , Y. Song , F. Wu , F. Lan , X. Hu , C. M. Cao , Y. Zhang , R. P. Xiao , Circulation. 2020, 142, 1077. 32677469 10.1161/CIRCULATIONAHA.119.044998

[7] T. C. Kuhn , J. Knobel , S. Burkert‐Rettenmaier , X. Li , I. S. Meyer , A. Jungmann , F. Sicklinger , J. Backs , F. Lasitschka , O. J. Müller , H. A. Katus , J. Krijgsveld , F. Leuschner , Circulation. 2020, 141, 1628. 32100557 10.1161/CIRCULATIONAHA.119.044914

[8] H. Cheng , S. Chang , R. Xu , L. Chen , X. Song , J. Wu , J. Qian , Y. Zou , J. Ma , Stem Cell Res. Ther. 2020, 11, 224. 32513270 10.1186/s13287-020-01737-0 PMC7278138

[9] L. P. Zhu , T. Tian , J. Y. Wang , J. N. He , T. Chen , M. Pan , L. Xu , H. X. Zhang , X. T. Qiu , C. C. Li , K. K. Wang , H. Shen , G. G. Zhang , Y. P. Bai , Theranostics. 2018, 8, 6163. 30613290 10.7150/thno.28021 PMC6299684

[10] E. A. Blackwood , D. J. Thuerauf , M. Stastna , H. Stephens , Z. Sand , A. Pentoney , K. Azizi , T. Jakobi , J. E. Van Eyk , H. A. Katus , C. C. Glembotski , S. Doroudgar , J. Mol. Cell Cardiol. 2020, 143, 132. 32339566 10.1016/j.yjmcc.2020.04.012 PMC8597053

[11] S. Kumar , G. Wang , N. Zheng , W. Cheng , K. Ouyang , H. Lin , Y. Liao , J. Liu , Hypertension. 2019, 73, 1058. 30827145 10.1161/HYPERTENSIONAHA.118.12267

[12] H. Shi , Y. Gao , Z. Dong , J. Yang , R. Gao , X. Li , S. Zhang , L. Ma , X. Sun , Z. Wang , F. Zhang , K. Hu , A. Sun , J. Ge , Circ. Res. 2021, 129, 383. 34015941 10.1161/CIRCRESAHA.120.318629 PMC8291144

[13] D. Zhu , S. Liu , K. Huang , Z. Wang , S. Hu , J. Li , Z. Li , K. Cheng , Circ. Res. 2022, 131, e135. 36252111 10.1161/CIRCRESAHA.122.321384 PMC9667926

[14] J. Kim , J. S. Won , A. K. Singh , A. K. Sharma , I. Singh , Antioxid. Redox Signal. 2014, 20, 2514. 24063605 10.1089/ars.2013.5223 PMC4026100

[15] G. Wei , C. Li , X. Jia , J. Xie , Z. Tang , M. Jin , Q. Chen , Y. Sun , S. He , X. Li , Y. Chen , H. Zheng , W. Liao , Y. Liao , J. Bin , S. Huang , J. Adv. Res. 2023, 53, 199. 36587763 10.1016/j.jare.2022.12.014 PMC10658329

[16] P. C. Dinh , D. Paudel , H. Brochu , K. D. Popowski , M. C. Gracieux , J. Cores , K. Huang , M. T. Hensley , E. Harrell , A. C. Vandergriff , A. K. George , R. T. Barrio , S. Hu , T. A. Allen , K. Blackburn , T. G. Caranasos , X. Peng , L. V. Schnabel , K. B. Adler , L. J. Lobo , M. B. Goshe , K. Cheng , Nat. Commun. 2020, 11, 1064. 32111836 10.1038/s41467-020-14344-7 PMC7048814

[17] K. A. Kaminski , T. A. Bonda , J. Korecki , W. J. Musial , Int. J. Cardiol. 2002, 86, 41. 12243849 10.1016/s0167-5273(02)00189-4

[18] M. Horckmans , L. Ring , J. Duchene , D. Santovito , M. J. Schloss , M. Drechsler , C. Weber , O. Soehnlein , S. Steffens , Eur. Heart J. 2017, 38, 187. 28158426 10.1093/eurheartj/ehw002

[19] X. Zhao , S. M. Ting , C. H. Liu , G. Sun , M. Kruzel , M. Roy‐O'Reilly , J. Aronowski , Nat. Commun. 2017, 8, 602. 28928459 10.1038/s41467-017-00770-7 PMC5605643

[20] K. Ishibashi , M. Imai , Am J. Physiol Renal Physiol. 2002, 282, F367. 11832417 10.1152/ajprenal.00364.2000

[21] A. Y. Law , L. Y. Ching , K. P. Lai , C. K. Wong , Mol. Cell. Endocrinol. 2010, 314, 118. 19628018 10.1016/j.mce.2009.07.007

[22] C. W. Lee , I. Hwang , C. S. Park , H. Lee , D. W. Park , S. J. Kang , S. W. Lee , Y. H. Kim , S. W. Park , S. J. Park , J. Clin. Pathol. 2013, 66, 787. 23757035 10.1136/jclinpath-2013-201563

[23] M. Y. Chan , M. Efthymios , S. H. Tan , J. W. Pickering , R. Troughton , C. Pemberton , H. H. Ho , J. F. Prabath , C. L. Drum , L. H. Ling , W. M. Soo , S. C. Chai , A. Fong , Y. Y. Oon , J. P. Loh , C. H. Lee , R. S. Y. Foo , M. A. Ackers‐Johnson , A. Pilbrow , A. M. Richards , Circulation. 2020, 142, 1408. 32885678 10.1161/CIRCULATIONAHA.119.045158 PMC7547904

[24] X. Jiang , D. Zhao , L. J. Bao , Eur. Rev. Med. Pharmacol. Sci. 2022, 26, 4309. 35776032 10.26355/eurrev_202206_29070

[25] A. Mohammadipoor , R. H. Lee , D. J. Prockop , T. J. Bartosh , Transl Res. 2016, 177, 127. 27469269 10.1016/j.trsl.2016.06.011 PMC5099094

[26] C. C. T. Leung , C. K. C. Wong , Transl. Oncol. 2021, 14, 100881. 33074126 10.1016/j.tranon.2020.100881 PMC7568195

[27] J. Sun , E. Murphy , Am. J. Physiol. Heart Circ. Physiol. 2010, 299, H1309. 20833954 10.1152/ajpheart.00373.2010 PMC2993222

[28] I. Dal Pra , A. Chiarini , E. F. Nemeth , U. Armato , J. F. Whitfield , J. Cell Biochem. 2005, 96, 428. 16052472 10.1002/jcb.20511

[29] B. Zhang , W. Crankshaw , R. Nesemeier , J. Patel , I. Nweze , J. Lakshmanan , B. G. Harbrecht , J. Surg. Res. 2015, 193, 795. 25150084 10.1016/j.jss.2014.07.042 PMC4268076

[30] S. Zhao , X. Tang , Z. Miao , Y. Chen , J. Cao , T. Song , D. You , Y. Zhong , Z. Lin , D. Wang , Z. Shi , X. Tang , D. Wang , S. Chen , L. Wang , A. Gu , F. Chen , L. Xie , Z. Huang , H. Wang , Y. Ji , Redox Biol. 2022, 52, 102290. 35334246 10.1016/j.redox.2022.102290 PMC8942817

[31] Y. Shu , C. Zou , Y. Cai , Q. He , X. Wu , H. Zhu , M. Qv , Y. Chao , C. Xu , L. Tang , X. Wu , Redox Biol. 2022, 56, 102420. 35969998 10.1016/j.redox.2022.102420 PMC9399387

[32] L. Luo , Y. Li , Z. Bao , D. Zhu , G. Chen , W. Li , Y. Xiao , Z. Wang , Y. Zhang , H. Liu , Y. Chen , Y. Liao , K. Cheng , Z. Li , Adv. Mater. 2024, 36, 2302686. 10.1002/adma.202302686 37665792

