# Supplementary material for: Hypoxia Microenvironment Preconditioning Attenuated Myocardial Ischemia‐Reperfusion Injury via Stc1‐Mediating Cardiomyocyte Self‐Protection and Neutrophil Polarization
Source: Adv Sci (Weinh). 2024 Dec 16;12(6):2411880. doi: 10.1002/advs.202411880 (PMC11809403; doi:10.1002/advs.202411880)
Supplement: Supplementary file 1 — Supporting Information [file ADVS-12-2411880-s001.docx]

Supporting Information

Hypoxia microenvironment preconditioning attenuated myocardial ischemia-reperfusion injury via Stc1-mediating cardiomyocyte self-protection and neutrophil polarization

Haoxiang Huang^#^, Yifei Ruan^#^, Chuling Li ^#^, Hao Zheng, Yating Tang, Yijin Chen, Fengling He, Yu Liu, Guangkai Wu, Zhenhua Li, Yuegang Wang, Yulin Liao, Jianping Bin, Yanmei Chen*

**Detailed Materials and methods**

**Cell culture and treatment**

Postnatal day-1 (P1) mice were anesthetized with 2% isoflurane and subjected to cervical dislocation. Cardiac tissue fragments from neonatal mice were digested with 0.25% trypsin (Invitrogen) at 4°C for 14 hours. Then, the tissue fragments were transferred into a digestion solution (1 mg/ml collagenase type II (Gibco) and 5 mg/ml bovine serum albumin (BSA, Sigma-Aldrich) in phosphate buffered saline (PBS)) and further digested by incubation for 20 minutes at 37°C with constant stirring. The cell suspension was then collected and centrifuged to separate the cells. Subsequently, the cells were resuspended in Dulbecco’s modified Eagle medium/Nutrient Mixture F12 (DMEM/F12) medium supplemented with 10% fetal bovine serum (FBS) at 37°C, and 21% O2/5% CO2. After 2 hours, the non-adherent cardiomyocytes were plated in DMEM/F12 containing 10% FBS and 1% penicillin/streptomycin. The concentration of rStc1 and nps2143 used for cardiomyocytes intervention were 100ng/ml and 5μg/ml, respectively.

Neutrophils were isolated from the mouse bone marrow using a Mouse Neutrophil Negative Selection Kit (Stemcell, 19762RF) according to the manufacturer’s instructions. Briefly, the bone marrow cells were flushed from the femur and tibia into RoboSep^TM^ Buffer using a syringe equipped with a 23-gauge needle. The clumps were dispersed by gently passing the cell suspension several times through a syringe. The remaining clumps and debris were removed by passing the cell suspension through a 70 μm mesh nylon strainer. Cells were centrifuged at 300 xg for 10 minutes and resuspended at 1 x 10^8 cells/ml in RoboSep^TM^ Buffer. Rat serum was added to the isolated bone marrow suspension, followed by an enrichment cocktail in a ratio of 50μl/ml. After the mixture was incubated on ice for 15 min, centrifuged at 300 xg for 10 minutes, the supernatant was removed. Resuspend cells using RoboSep^TM^ Buffer, add biotin reagent at a ratio of 50ul/ml, and incubate on ice for 15 minutes. Finally, add magnetic particles to label non-neutrophils and use magnets to remove non-neutrophils. The isolated neutrophils were cultured in RPMI 1640 medium containing 10% FBS and 1% penicillin/streptomycin at 37°C and 21% O2/5% CO2. The concentration of rStc1 and nps2143 used for neutrophils intervention were 60ng/ml and 3μg/ml, respectively.

**Myocardial Cas9 knockin transgenic mouse model**

The Shanghai Model Organisms Center, Inc. provided the Cre-dependent Cas9 knockin mouse model. The model was created via homologous recombination within JM8A3 embryonic stem (ES) cells and later implanted into C57BL/6J blastocysts, adhering to standard protocols. The construction process involved the design of a targeting vector that included a ubiquitously expressed CAG promoter, a loxP-flanked PGK-Neo-polyA sequence, and a Cas9 protein inserted into the first intron of the Rosa26 locus. The construct was linearized and subsequently electroporated into JM8A3 embryonic stem cells. PCR amplified both recombinant arms in the targeted single-ES cell colonies, which were then screened. Sequencing confirmed the correct insertion of the PCR products. Following correct targeting, colonies were injected into blastocysts, resulting in chimeric mice. Crossing the high-percentage chimeric males with C57BL/6J females produced heterozygous Cre-dependent Cas9 mice (Rosa26 LSL-Cas9-tdTomato). The Rosa26-LSL-Cas9-tdTomato mice were intercrossed to produce homozygous Rosa26-LSL-Cas9-tdTomato mice. These mice were crossed with α-MHC-Cre transgenic mice, thereby generating myocardial Rosa26-Cas9-tdTomato mice for later experiments. Adenovirus (Adv) vectors containing the green fluorescent protein (GFP) gene (Adv-GFP) for sg-CaSR, and sg-NC were synthesized by novoprotein (Suzhou, China). The sequence of sg-CaSR were as follows: (F) ACAGCCCGCTGAAGCAACGC; (R) GAGAGACCGAGGTCTAGCGC.

**Cell culture model of hypoxia/reoxygenation (H/R) injury**

For the hypoxia treatment, use sugar-free medium to culture cardiomyocytes or neutrophils and place the cells in a hypoxic incubator (Thermo Fisher Scientific) with 1% O2/94% N2/5% CO2 for 12 hours. Reoxygenation was achieved by replacing the sugar-free medium with DMEM/F12 containing 10% FBS and 1% penicillin/streptomycin and incubating the cells in a normal incubator (Thermo Fisher Scientific) with 21% O2/5% CO2 for 6 hours.

**Co-culture system construction**

The co-culture system is achieved through transwell chambers. Specifically, primary cardiomyocytes were seeded into the lower chamber of the Transwell chamber, and the separated neutrophils were placed in the higher chamber of the Transwell chamber.

**Recombinant adenovirus construction**

Recombinant adenovirus carrying FLAG-tagged Stat3-WT was purchased from Hanyi Biotechnology Co., Ltd. (Guangzhou, China). A single mutation from Cys259 to Ala (C259A; Hanyi Biotechnology Co., Ltd., Guangzhou, China) was confirmed by DNA sequencing. The sequences of the primers used for mutation were as follows (mutated bases in lower case): Stat3-C259A, CCCAACATCgccCTGGACCGT.

**AAV9 vectors injection in adult mice**

The AAV9 carrying wild-type Stat3 and its mutants (C259A) were synthesized by Hanyi Biotechnology Co., Ltd. (Guangzhou, China). Adult mice were anaesthetized and mechanically ventilated, after which the heart was exposed and intramyocardially injected with AAV9 vector at 5–6 sites with a dose of 1 × 10^11^ viral genome particles per animal (approximately 25 μl) by using an insulin syringe with a 30-gauge needle.

**Serum biomarker detection**

Blood samples were allowed to equilibrate at room temperature for 30 min before centrifugation at 1,000 ×g for 15 min. Subsequently, serum samples were collected and preserved at -80°C to facilitate subsequent analysis. Several key biomarkers in serum samples, including cardiac troponin T (cTnT, Mlbio, ml037292V), N-terminal prohormone of brain natriuretic peptide (NTproBNP, Mlbio, ml001918-2), creatine kinase-MB (CK-MB, Jinmei, JM-03084M2), and lactate dehydrogenase (LDH, Jinmei, JM-12891M2). They were quantified by using ELISA kits to estimate infarction size and extent.

**Measurement of infarct area and area at risk**

After 24 hours of reperfusion, each mouse was anesthetized, intubated and the chest was opened. The ascending aorta was cannulated and perfused with saline to remove blood. The left anterior descending coronary artery was occluded using a 6-0 surgical suture at the ligation site. To visualize the area at risk (AAR), 1% Evans blue dye was injected into the aorta. Hearts were excised and washed in phosphate-buffered saline (PBS). The tissues were frozen at -20°C for 1 hour, cross-sectioned at a thickness of 1 mm, and incubated in 1% TTC (Sangon Biotech Co. Ltd, Shanghai) at 37°C for 15 min. The infarct area (pale), AAR (red), and left ventricle area (LV) of each section were measured using ImageJ.

**Echocardiography**

Mice were anesthetized with 2% isoflurane and subjected to transthoracic echocardiography (VisualSonics VeVo 2100 Imaging System, Toronto, Canada) to assess cardiac structure and function. Body temperatures were maintained between 36.9°C and 37.3°C. M-mode tracings in the parasternal short-axis view were used to measure the left ventricular internal diameter at end-diastole (LVEDd) and end-systole (LVESd) that were used to calculate the left ventricular fractional shortening (LVFS) and left ventricular ejection fraction (LVEF).

**Microscopic imaging of cell death**

To observe the morphology of cardiomyocytes, the cells were seeded into 6-well plates for image capture. Propidium ioddied (PI; 1 µg/mL) was added to the culture medium to evaluate cell membrane integrity. Cell death was visualized by continuous live imaging using the CellSens Dimension platform. Static bright-field and fluorescent images of the cells were captured using an Olympus IX71 confocal microscope. All imaging data are representative of at least three randomly selected fields.

**Annexin V-FITC/PI staining**

The apoptosis ratio of CMs was assessed using the Annexin V-FITC/PI apoptosis detection kit (MultiSciences Biotech Co.,Ltd, Hanzhou), according to the manufacturer's instructions. Briefly, the cardiomyocytes were resuspended in 0.1 mL of binding buffer and then incubated with Annexin V-FITC/PI buffer in the dark for 10 min. Fluorescence intensity was determined using flow cytometry (FACScan, BD Biosciences), and data were analyzed using FlowJo 7.6.1 software.

**TUNEL staining**

We used TUNEL staining kit for TUNEL staining, according to the manufacturer's instructions (KeyGen Biotech Co.,Ltd, Nanjing). Briefly, after washing the intervened cardiomyocytes with PBS, fix the cells with 4% paraformaldehyde for 30 minutes, add 0.1% Triton X-100 to PBS and incubate at room temperature for 5 minutes. After washing, add TUNEL detection solution and incubate at 37 ° C in the dark for 1 hour. Wash the cells three times with PBS and observe and take photos under a fluorescence microscope.

**Flow cytometry of neutrophil phenotypes**

After cell fixation and membrane rupture, single-cell suspensions isolated from the LV or neutrophils isolated from the bone marrow were blocked with 5% heat-inactivated mouse serum (M5905; Sigma) and incubated with APC anti-Ly6G antibody (BioLegend) and Alexa Fluor 700 anti-CD206 antibody (BioLegend). Flow cytometry experiments were performed using a FACScan (BD Biosciences) and the data were analyzed using FlowJo 7.6.1 software. N1 neutrophils were defined as Ly-6G^+^CD206^-^ and N2 neutrophils were defined as Ly-6G^+^CD206^+^ cells.

**Determination of dissociation constant (Kd)**

We use the saturation concentration method to determine by measuring the concentration of free R, free L, and R-L complex in the solution after dynamic equilibrium. The specific calculation formula is:

$$\left[ \boldsymbol{RL} \right]\boldsymbol{=}\left[ \boldsymbol{R} \right]\boldsymbol{t\times}\left( \frac{\left[ \boldsymbol{L} \right]\boldsymbol{t}}{\boldsymbol{Kd+}\left[ \boldsymbol{L} \right]\boldsymbol{t}} \right)$$

[R]=Concentration of free R, [L]=concentration of free L, [R+L] =concentration of R-L complex

**MPO, MMP9 detection of neutrophil supernatant**

Neutrophil supernatant concentration of MPO and MMP9 were determined using the CSB-E08723m (Cusabio), and CSB-E08007m (Cusabio) ELISA kits respectively, according to the manufacturers’ instructions.

**Trypan blue staining**

The adherent cells were digested into a single-cell suspension and diluted to the required concentration. A small drop of freshly prepared dye solution was added to every 0.1 mL of cell suspension and incubated at room temperature for 3-5 minutes. A blood cell counting board was placed, and the count was observed under a high-power microscope. Dead cells were light blue, enlarged, and dull; living cells were not colored, maintained their normal shape, and were shiny.

**Immunostaining and immunofluorescence analysis**

Heart sections (5 μm) and cultured cells were fixed with 4% paraformaldehyde for 30 minutes. Then, the samples were permeabilized with 1% Triton X-100 (Biosharp, China) in PBS for 10 minutes, blocked with 1% BSA in PBS for 1 hour at room temperature and incubated with the primary antibodies diluted in 1% BSA blocking solution at 4°C overnight. Then, the heart sections and cultured cells were washed three times with PBS and incubated with secondary antibodies for 1 hour at room temperature followed by 20 minutes of DAPI staining to visualize the cell nuclei. MitoSOX (Invitrogen, M36008) was used to detect mitochondria-derived ROS. DCFH-DA fluorescent probe (Bestbio Biotech Co.,Ltd, Shanghai) was used to detect overall ROS *in vitro*. The stained sections and cells were imaged with a Leica (TCS Sp8) confocal microscope. The primary and secondary antibodies were listed in Supplementary Table 3.

**RNA isolation and qRT-PCR**

Total RNA was extracted from the isolated CMs or mouse heart tissue samples using a Total RNA Kit II (Omega), according to the manufacturer’s instructions. A PrimeScript™ RT reagent Kit (TaKaRa Bio) was used to perform reverse transcription to synthesize single-stranded cDNA. Quantitative reverse transcriptase-polymerase chain reaction (qRT-PCR) was performed with the SYBR Green PCR Master Mix (TaKaRa Bio) using a LightCycler480 (Roche). The primers used for qRT-PCR analysis are listed in Supplementary Table 1.

**Western blotting**

Total cell lysates were isolated using radio-immunoprecipitation assay (RIPA) buffer supplemented with 1:100 protease (Sigma, P8340) and 1:100 phosphatase inhibitor cocktails (Sigma, P5726, and P0044). Protein concentrations were quantified using bicinchoninic acid assay (Thermo Fisher, 23225). The lysates were separated by 8-12% SDS-PAGE and transferred onto polyvinylidene difluoride membranes (Millipore). Membranes were blocked and probed overnight with primary antibodies. The membranes were then incubated with secondary antibody for 1 hour at room temperature. The primary and secondary antibodies are listed in Supplementary Table 2. The results were visualized using an Odyssey detection system (LI-COR Biosciences, Lincoln, NE, USA), and ImageJ software was used to calculate the relative density of the proteins.

**RNA-seq**

Total RNA was extracted using the TRIZOL reagent (Thermo Fisher, Catalog#5596018, USA). After precipitation, the RNA samples were sent to LC Science for bulk RNA sequencing. RNA-seq analysis was performed using the RStudio software (Version 1.4.1717). Filtration and normalization of the lowly expressed genes were performed using the edge R package. Lowly expressed genes were filtered as unexpressed if the average count per million (CPM) was <0.5. Differential expression was analyzed using the Limma package and counts were transformed into log2CPM values using the voom function. Subsequently, an interactive plot of the differentially expressed genes was acquired via the glMDPlot function in the Glimma package, and the subset of genes of interest across the groups was selected for further analysis.

**Biotin switch assay**

S-nitrosylation of Stat3 (SNO-Stat3) was detected by using a biotin switch assay with the S-nitrosylation Protein Detection Assay Kit (Cayman Chemical) as described previously.^1^ Briefly, cell lysates were incubated with blocking buffer for 30 min to block free thiols; proteins were then precipitated with cold acetone at −20°C for 1 h. Then, the S-nitrosothiols were reduced to free thiols with reducing buffer and labeled with biotin. Samples incubated without reducing buffer were used as negative controls. The biotinylated proteins were purified by incubating with avidin-coupled agarose beads (Thermo Scientific) overnight at 4°C. The SNO-Stat3 levels were detected by western blotting with anti-Stat3 antibody.

**Co-immunoprecipitation (Co-IP) assay**

Co-IP assays were performed according to a previous study.^[2]^ Briefly, cell lysates were collected and incubated with antibodies at 4°C for 12 hours. Then, they were incubated with protein A/G magnetic beads (Thermo Scientific, USA). Finally, the beads were collected and subjected to western blotting.

**Single cell RNA sequencing (scRNA-seq) Data Analysis**

**（1）Data acquisition and code availability**

The publicly available single-cell transcriptomes at day 1 following ischemia-reperfusion injury in adult mice were acquired from the NCBI GEO database (GSE146285).

**（2）Quality control and normalization**

In this study, we used the R package Seurat software (version 4.0.2) to conduct an in-depth analysis of the single-nucleus gene expression matrix. To eliminate low-quality cells and reduce interference, this analysis began by implementing stringent quality control measures, in which low-quality nuclei were excluded based on specific criteria, including UMI counts, expressed gene counts, and the proportion of mitochondrial transcripts. Following this quality filtering step, the remaining cells underwent normalization with the "LogNormalize" method in the NormalizeData function. The top 2000 highly variable genes were selected using the FindVariableFeatures function with the "vst" method. The ScaleData function was used for subsequent calculations of the normalized data.

**（3）Unsupervised clustering analysis and cell annotation**

Highly variable genes were used for Principal Component Analysis (PCA). To reduce batch effects and integrate scaled data, we employed the Harmony R software package. Unsupervised cell clustering was performed by considering the top 20 principal components using the Seurat functions, FindNeighbors, and FindClusters. For data visualization, cells were projected onto a 2D space based on aligned canonical correlation analysis using t-distributed Stochastic Neighbor Embedding (tSNE). To identify the cluster-specific genes, we applied the FindAllMarkers function using the Wilcoxon rank-sum test in Seurat. Manual annotation of the cell types was performed using highly specific marker genes associated with each cluster.

**（4）Cellular communication analysis**

To elucidate the interactions among different cell types, CellChat Score, an open-source R package software, was used to perform the inference, visualization, and analysis of intercellular communication of scRNA-seq data. This process involved identifying the expressed ligands and receptors in single-cell data, along with their interactions, projecting gene expression data onto the protein-protein interaction (PPI) network, calculating the communication probability, and inferring the CellChat network. Cell interactions were visualized using the netVisualcircle function.

**NO detection**

We used Nitric oxide (NO) content detection kit for NO detection, according to the manufacturer's instructions (Solarbio). Briefly, add the extraction solution in a ratio of 500-1000:1 based on the number of cells (10^4^): volume of extraction solution (mL), then sonicate the cells in an ice bath (power 200w, sonication for 3 seconds, interval for 7 seconds, total time for 5 minutes), then centrifuge at 4 ℃, 12000rpm for 15 minutes, discard the precipitate, and take the supernatant and place it on ice for testing. Preheat the spectrophotometer for at least 30 minutes and adjust the wavelength to 550nm for detection.

**Molecular docking**

The online HDOCK Server (Hust.edu.cn) was used to conduct simulation docking research on protein Stc1 and protein CaSR by utilizing PyMOL (version 4.3.0) software（<https://pymol.org/>) that is used for pre docking processing and visual display of docking compounds. Discovery Studio (Discovery Studio Visualizer v4.5.0.15071) software was used for force analysis.

**Molecular dynamics simulation**

The crystal structure corresponding to the full-length sequence of the Stat3 protein was predicted using AlphaFold2, and the S-nitrosylation mutation of Cys259 was performed using the Mutate Residue module in the Schrödinger software to generate two protein crystal files before and after the mutation. The two protein crystals obtained were processed by protein preprocessing to regenerate the native ligand states, carry out H-bond assignment optimization, protein energy minimization, and water removal using the Protein Preparation Wizard module of Schrödinger software. The Schrödinger interaction module was used to display the interaction between the phosphorylation sites Y705 and S727 and their nearby residues before and after the mutation, and the Measure module was used to measure the distance of the interaction.

**ADA detection**

We used SMC™ Immunogenicity Bead Based Assay Development Kit for ADA detection, according to the manufacturer's instructions. Briefly, the process began with coating the beads with the target protein or peptide and then adding the serum samples containing the potential ADA. After an incubation period, the ADA would bind to the coated beads. The unbound components were then washed away. Next, a detection reagent was added that would bind specifically to the ADA, allowing for their quantification. The fluorescence generated by this binding was then measured using a flow cytometer, providing a quantitative assessment of the ADA in the serum samples.

**Biodistribution of extracellular vesicles after intrapericardial injection**

Cy7 (Cyanine 7) is a fluorescent reagent commonly used for protein labeling.^[3]^ The recombinant protein Stc1 was labeled by dialysis. After an ischemic event and before reperfusion injury occurred, Cy7-rStc1 in 20 μL PBS or HA hydrogel were injected into the pericardial cavity. Images were acquired at 0, 6, 12, 24, and 48 hours after Cy7-rStc1 injection. Aura software was used to calculate the relative density of the fluorescence signal.

**References**

[1] Zhao S, Tang X, Miao Z, Chen Y, Cao J, Song T, You D, Zhong Y, Lin Z, Wang D, Shi Z, Tang X, Wang D, Chen S, Wang L, Gu A, Chen F, Xie L, Huang Z, Wang H, Ji Y. Hsp90 S-nitrosylation at Cys521, as a conformational switch, modulates cycling of Hsp90-AHA1-CDC37 chaperone machine to aggravate atherosclerosis. *Redox Biol*. **2022** Jun; wo52:102290.

[2] Chen Y, Xu T, Li M, Li C, Ma Y, Chen G, Sun Y, Zheng H, Wu G, Liao W, Liao Y, Chen Y, Bin J. Inhibition of SENP2-mediated Akt deSUMOylation promotes cardiac regeneration via activating Akt pathway. *Clin Sci (Lond)*. **2021** Mar 26;135(6):811-828.

[3] Lim B, Yao Y, Huang AL, Yap ML, Flierl U, Palasubramaniam J, Zaldivia MTK, Wang X, Peter K. A Unique Recombinant Fluoroprobe Targeting Activated Platelets Allows In Vivo Detection of Arterial Thrombosis and Pulmonary Embolism Using a Novel Three-Dimensional Fluorescence Emission Computed Tomography (FLECT) Technology. *Theranostics*. **2017** Feb 26;7(5):1047-1061.

**Supplemental Tables**

**Supplementary Table S1: The primers used for qPCR in this study.**

| Primers name |  | Sequence (5’-3’) |
| --- | --- | --- |
| Bax | +  - | AGACAGGGGCCTTTTTGCTAC  AATTCGCCGGAGACACTCG’ |
| Bcl2 | +  - | GTCGCTACCGTCGTGACTTC  CAGACATGCACCTACCCAGC |
| GSDMD | +  - | CCATCGGCCTTTGAGAAAGTG  ACACATGAATAACGGGGTTTCC |
| Caspase11 | +  - | ACAAACACCCTGACAAACCAC  CACTGCGTTCAGCATTGTTAAA |
| Gpx4 | +  - | GATGGAGCCCATTCCTGAACC  CCCTGTACTTATCCAGGCAGA |
| ACSL4 | +  - | CTCACCATTATATTGCTGCCTGT  TCTCTTTGCCATAGCGTTTTTCT |
| RIPK3 | +  - | TCTGTCAAGTTATGGCCTACTGG  GGAACACGACTCCGAACCC |
| RIPK1 | +  - | GAAGACAGACCTAGACAGCGG  CCAGTAGCTTCACCACTCGAC |
| Ccl3 | +  - | TTCTCTGTACCATGACACTCTGC  CGTGGAATCTTCCGGCTGTAG |
| Ccl5 | +  - | GCTGCTTTGCCTACCTCTCC TCGAGTGACAAACACGACTGC |
| IL12α | +  - | CTGTGCCTTGGTAGCATCTATG  GCAGAGTCTCGCCATTATGATTC |
| Arg1 | +  - | CTCCAAGCCAAAGTCCTTAGAG  AGGAGCTGTCATTAGGGACATC |
| CD206 | +  - | CTCTGTTCAGCTATTGGACGC  CGGAATTTCTGGGATTCAGCTTC |
| Tgfβ1 | +  - | CTCCCGTGGCTTCTAGTGC  GCCTTAGTTTGGACAGGATCTG |

**Supplementary Table S2: Antibodies for western blots and co-immunoprecipitation assay.**

|  | Vendor or  Source | Catalog # | Dilute  Proportion |
| --- | --- | --- | --- |
| anti-Arg1 | Proteintech | 16001-1-AP | 1:5000-1:50000 |
| anti-CD206 | Proteintech | 18704-1-AP | 1:500-1:1000 |
| anti-α Tubulin | Proteintech | 11224-1-AP | 1:2000-1:15000 |
| anti-Stc1 | Proteintech | 20621-1-AP | 1:500-1:1000 |
| anti-B4galt1 | Abcam | ab121326 | 1:250-1:2500 |
| anti-Sephinh1 | Thermo Fisher | PA5-120817 | 1:500-1:2000 |
| anti-Bmp3 | Abcam | ab134724 | 1:500-1:1000 |
| anti-Lama5 | Abcam | ab184330 | 1:1000 |
| anti-Olfm2 | Abcam | ab154196 | 1:1000-1:10000 |
| anti-Albumin | Proteintech | 16475-1-AP | 1:5000-1:50000 |
| anti-Bax | Proteintech | 50599-2-Ig | 1:2000-1:16000 |
| anti-Bcl2 | Proteintech | 26593-1-AP | 1:1500 |
| anti-Cleaved caspase 3 | Abcam | ab214430 | 1:5000 |
| anti-Caspase 3 | Proteintech | 19677-1-AP | 1:500-1:2000 |
| anti-Cleaved GSDMD-N | CST | 10137 | 1:1000 |
| anti-GSDMD | Proteintech | 20770-1-AP | 1:2000-1:10000 |
| anti-Caspase 11 | Abcam | ab180673 | 1:1000 |
| anti-GAPDH | Proteintech | 10494-1-AP | 1:5000-1:40000 |
| anti-Flag | Proteintech | 20543-1-AP | 1:20000-1:100000 |
| anti-HA | Proteintech | 51064-2-AP | 1:5000-1:10000 |
| anti-Stat3 | Abcam | ab68153 | 1:1000-1:2000 |
| anti-pStat3(Ser727) | Abcam | ab32143 | 1:1000-1:10000 |
| anti-NOS2 | Proteintech | 22226-1-AP | 1:200-1:1000 |
| donkey anti-rabbit IgG H&L | Abcam | ab175772 | 1:10000 |
| Goat anti-Mouse IgG H&L | Abcam | ab6708 | 1:10000 |

**Supplementary Table S3: Antibodies for Immunofluorescence staining.**

|  | Vendor or  Source | Catalog # | Dilute  Proportion |
| --- | --- | --- | --- |
| anti- cTnT | Santa Cruz Biotechnology | sc-20025 | 1:50 |
| anti-GSDMD | Proteintech | 20770-1-AP | 1:50-1:500 |
| anti-CD3 | Proteintech | 17617-1-AP | 1:1000 |
| anti-F4/80 | CST | 70076S | 1:400 |
| anti-Ly6G | Invitrogen | RB6-8C5 | 1:100 |
| anti-CD206 | Proteintech | 18704-1-AP | 1:100 |
| anti-Stc1 | Santa Cruz | sc-293435 | 1:50-1:500 |
| goat anti-mouse IgG/Alexa Fluor 488 | Bioss | bs-0296G-AF488 | 1:100 |
| goat anti-rabbit IgG/Alexa Fluor 555 | Bioss | bs-0295G-AF555 | 1:100 |
| goat anti-mouse IgG/Alexa Fluor 647 | Bioss | bs-0296G-AF647 | 1:100 |
| goat anti-rabbit IgG/Alexa Fluor 647 | Bioss | bs-0295G-AF647 | 1:100 |

**Supplementary Table S4: Demographic statistics data of serum samples of patients**

| **Variables** | **Crude (n = 750)** |
| --- | --- |
| **Age (years), median (q25–q75)** | **70 (63-78)** |
| **BMI (kg/m^2^), median (q25–q75)** | **26.4463 (23.9390-29.4076)** |
| **Obesity (BMI ≥ 30 kg/m^2^), n (%)** | **160 (21.8)** |
| **Male, n (%)** | **519 (69.2)** |
| **Race, n (%)**  **European**  **Other** | **645 (86.0)**  **105 (14.0)** |
| **Smoking status,** **n (%)**  **Current-smoker**  **Ever-smoker** | **46 (6.1)**  **419 (55.9)** |
| **Non-smoker** | **285 (38.0)** |

**q25, 25th quantile; q75, 75th quantile.**

**BMI, body mass index.**

**
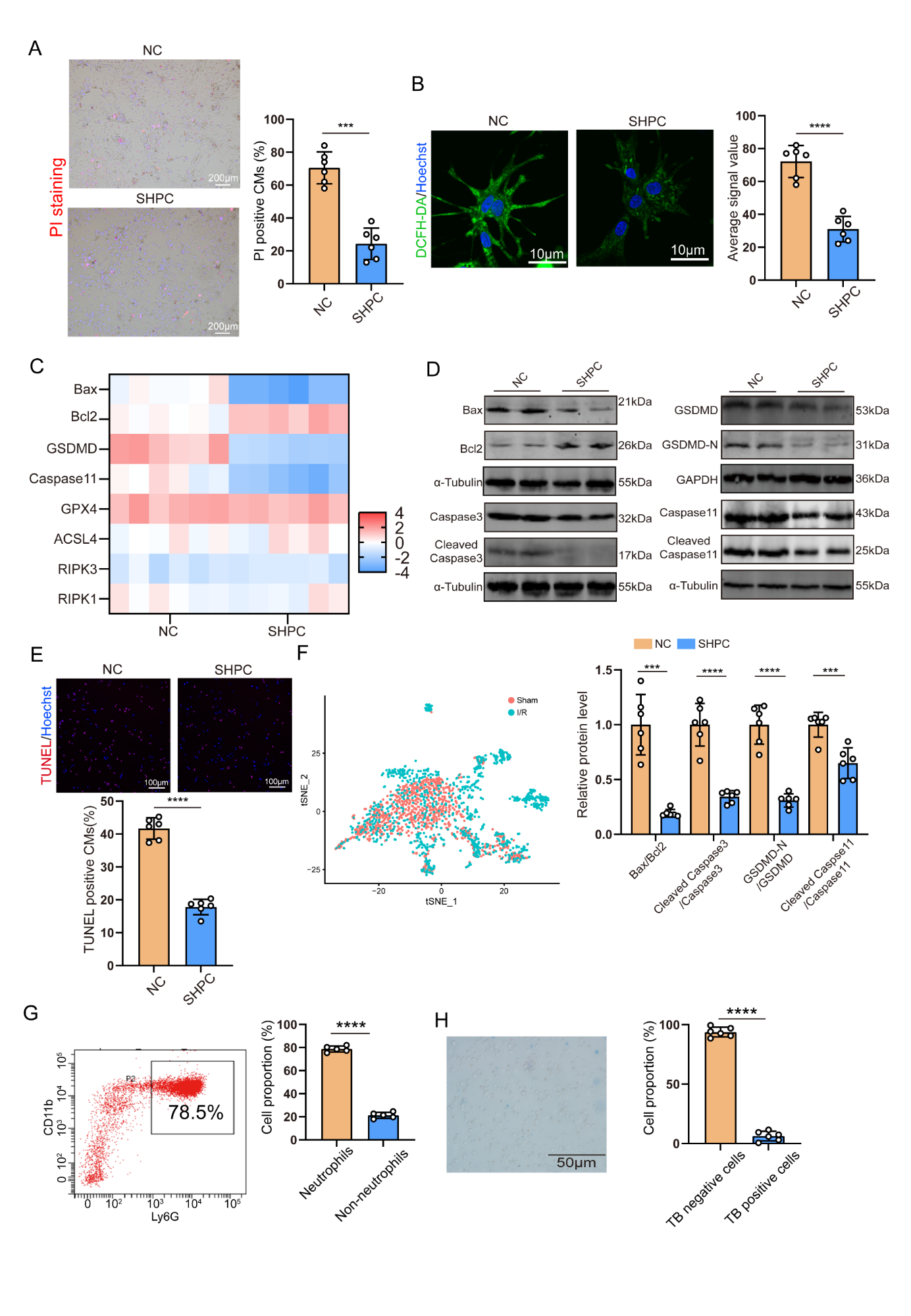
**

**Supplemental Figures and Figure Legends**

**Figure S1: The secretome of hypoxia preconditioned cardiomyocytes (SHPC) attenuated cardiomyocyte damage via inhibition of cardiomyocyte apoptosis and pyroptosis.**

(A) Cell viability measured by PI staining (1384 cardiomyocytes from 6 mice in the NC group and 1497 cardiomyocytes from 6 mice in the SHPC group; scale bar=200μm). (B) DCFH-DA staining in NC- or SHPC- treated cardiomyocytes after H/R treatment (182 cardiomyocytes from 6 mice in the NC group and 164 cardiomyocytes from 6 mice in the SHPC group; scale bar=10μm). (C) Bax, Bcl2, GSDMD, Caspase11, GPX4, ACSL4, RIPK3 and RIPK1 mRNA levels in NC- or SHPC-treated cardiomyocytes after H/R treatment (n=6 cell samples). (D) Bax, Bcl2, Cleaved caspase 3, Caspase 3, GSDMD-N, GSDMD, Cleaved caspase 11 and Caspase11 protein levels in NC- or SHPC-treated cardiomyocytes after H/R treatment (n=6 cell samples). (E) TUNEL staining in NC- or SHPC- treated cardiomyocytes after H/R treatment (1012 cardiomyocytes from 6 mice in the NC group and 1103 cardiomyocytes from 6 mice in the SHPC group; scale bar=100μm). (F) Single-cell RNA sequencing (scRNA-seq) data integration of sham and I/R heart tissue. (G) Flow cytometry results showing the purity of isolated neutrophils (n=6 cell samples). (H) Trypan blue staining showing cell viability of isolated neutrophils (1162 neutrophils from 6 mice; scale bar=50μm). Statistical significance was calculated using an unpaired t-test in A–F, H-I; ***P<0.001, and ****P<0.0001.

**
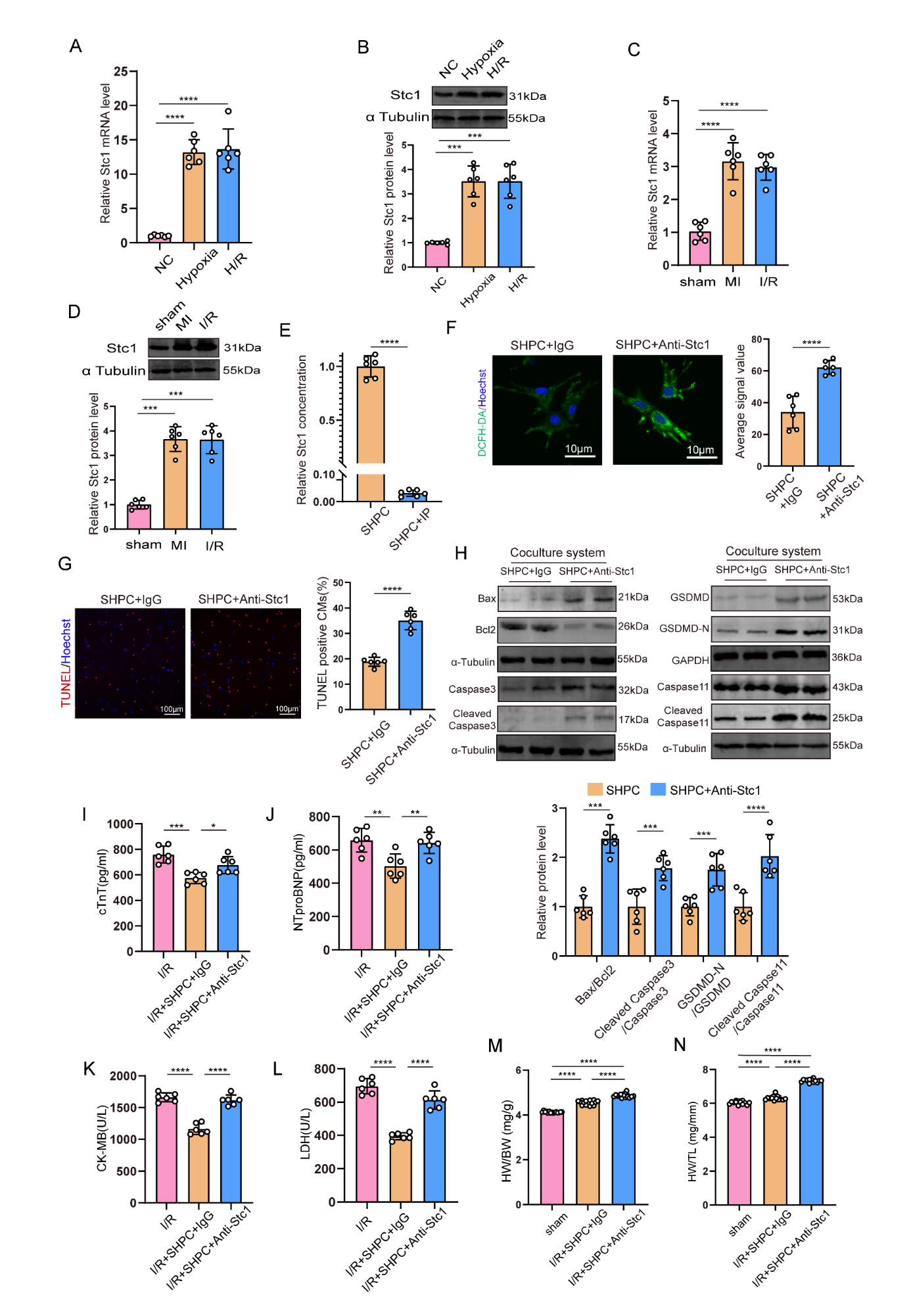
**

**Figure S2. Stc1 was the key factor inhibiting cardiomyocyte apoptosis and pyroptosis in secretome of hypoxia preconditioned cardiomyocytes (SHPC).**

(A) Stc1 mRNA levels in cardiomyocytes after hypoxia or H/R treatment (n=6 cell samples). (B) Stc1 protein levels in cardiomyocytes after hypoxia or H/R treatment (n=6 cell samples). (C) Stc1 mRNA levels in cardiomyocytes after hypoxia or H/R treatment (n=6 mice). (D) Stc1 protein levels in mouse hearts from sham, myocardial infarction (MI), ischemia/reperfusion (I/R) models (n=6 mice). (E) ELISA assay results showing the levels of Stc1 in cardiomyocyte lysate with or without IgG immunoprecipitation to bind Stc1 antibody (n=6 supernatant samples). (F) DCFH-DA staining in SHPC+IgG- or SHPC+Anti-Stc1- treated cardiomyocytes after H/R treatment (177 cardiomyocytes from 6 mice in the SHPC+IgG group and 181 cardiomyocytes from 6 mice in the SHPC+Anti-Stc1 group; scale bar=10μm). (G) TUNEL staining in SHPC+IgG- or SHPC+Anti-Stc1- treated cardiomyocytes after H/R treatment (923 cardiomyocytes from 6 mice in the SHPC+IgG group and 1002 cardiomyocytes from 6 mice in the SHPC+Anti-Stc1 group; scale bar=100μm). (H) Bax, Bcl2, Cleaved caspase 3, Caspase 3, Cleaved GSDMD-N, GSDMD, Cleaved caspase 11 and Caspase11 protein levels in cardiomyocytes co-cultured with SHPC+IgG- or SHPC+Anti-Stc1-treated neutrophils (n=6 cell samples). (I-L) Circulating levels of cTnT, NTproBNP, CK-MB and LDH in mice with SHPC+IgG or SHPC+Anti-Stc1 injection 24 hours after I/R surgery (n=6 mice). (M-N) The heart weight (HW)/body weight (BW) ratio and heart weight (HW)/tibial length (TL) ratio at 4 weeks after I/R surgery (n=12 mice). Statistical significance was calculated using one-way ANOVA in A-D, and H-N, an unpaired t-test in E-G; *P<0.05, **P<0.01, ***P<0.001, and ****P<0.0001.

**
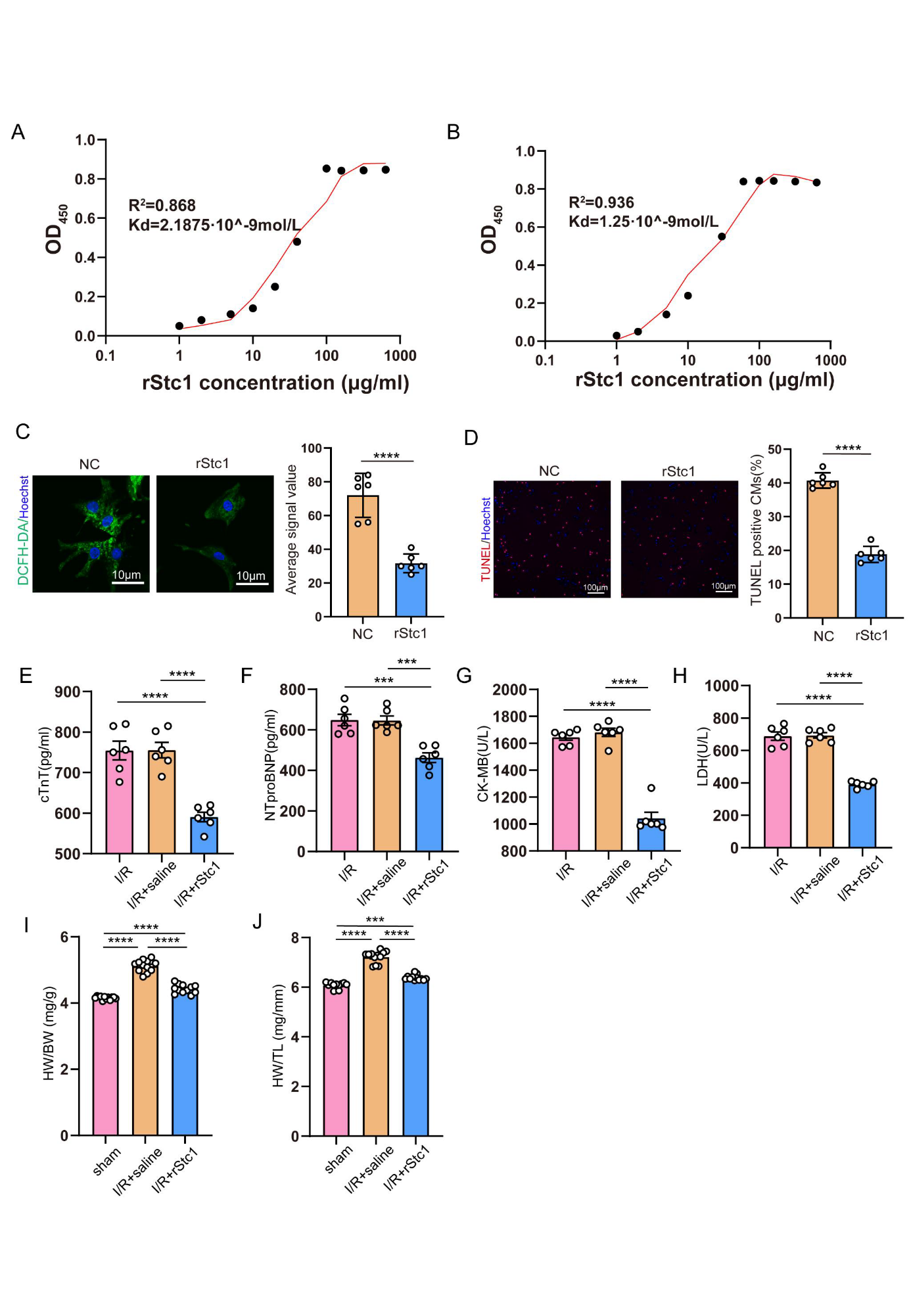
**

**Figure S3. Recombinant Stc1 significantly attenuated cardiac ischemia-reperfusion (I/R) injury via cardiomyocyte self-protection and modulation of neutrophil polarization.**

(A-B) The titration ELISA assay results showing the levels of Stc1 in cardiomyocyte and neutrophils supernatant with different rStc1 concentration intervention. (n=3 cell samples). (C) DCFH-DA staining in SHPC+IgG- or SHPC+Anti-Stc1- treated cardiomyocytes after H/R treatment (167 cardiomyocytes from 6 mice in the NC group and 174 cardiomyocytes from 6 mice in the rStc1 group; scale bar=10μm). (D) TUNEL staining in SHPC+IgG- or SHPC+Anti-Stc1- treated cardiomyocytes after H/R treatment (917 cardiomyocytes from 6 mice in the NC group and 889 cardiomyocytes from 6 mice in the rStc1 group; scale bar=100μm). (E-H) Circulating levels of cTnT, NTproBNP, CK-MB and LDH in mice with saline or rStc1 injection 24 hours after I/R surgery (n = 6 mice). (I-J) The heart weight (HW)/body weight (BW) ratio and heart weight (HW)/tibial length (TL) ratio at 4 weeks after I/R surgery (n=12 mice). Statistical significance was calculated using an unpaired t-test in C-D, one-way ANOVA in E-J; *P<0.05, **P<0.01, ***P<0.001, and ****P<0.0001.

**
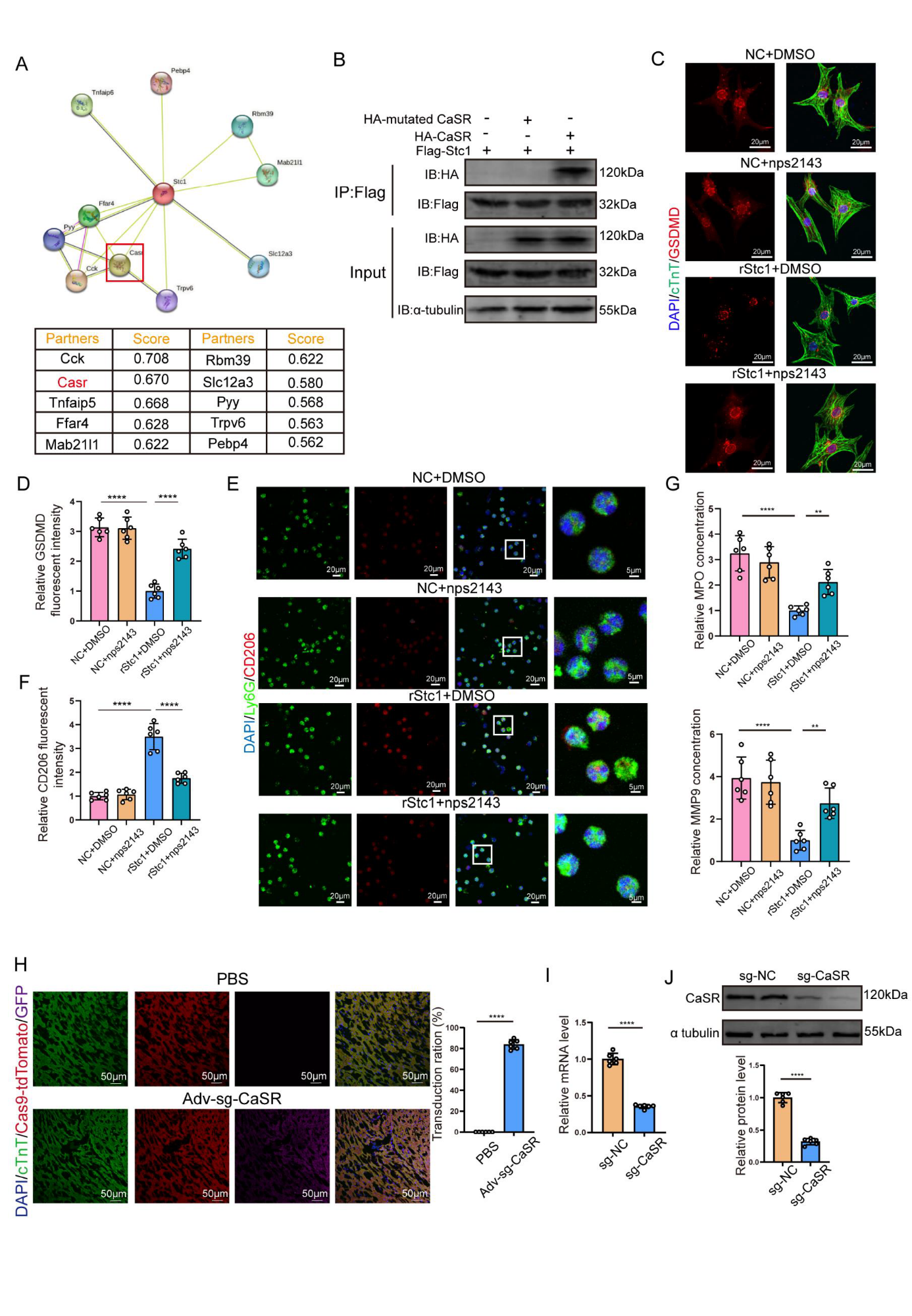
**

**Figure S4. Stc1 mediated cardioprotective effects are mediated through the CaSR.**

(A) Protein-protein interaction (PPI) network analysis of Stc1. (B) Stc1-CaSR co-immunoprecipitation assays using neutrophils transfected with HA-CaSR and Flag-Stc1 constructs individually or together. (C-D) Immunofluorescent staining for GSDMD (red) and cTnT (green) in NC+DMSO, NC+nps2143, rStc1+DMSO or rStc1+nps2143 group after H/R treatment (161 cardiomyocytes from 6 mice in the NC+DMSO group, 153 cardiomyocytes from 6 mice in the NC+nps2143 group, 137 cardiomyocytes from 6 mice in the rStc1+DMSO group and 156 cardiomyocytes from 6 mice in the rStc1+nps2143 group; scale bar=20μm). (E-F) NC+DMSO, NC+nps2143, rStc1+DMSO or rStc1+nps2143 group neutrophils were collected for immunofluorescent staining for Ly6G (green) and CD206 (red) after H/R treatment (388 neutrophils from 6 mice in the NC+DMSO group, 412 neutrophils from 6 mice in the NC+nps2143 group, 394 neutrophils from 6 mice in the rStc1+DMSO group and 422 neutrophils from 6 mice in the rStc1+nps2143 group; scale bar=20 μm [three on the left], and =5μm [rightmost]). (G) ELISA results showing concentrations of MPO and MMP9 in NC+DMSO, NC+nps2143, rStc1+DMSO or rStc1+nps2143 group neutrophil supernatants after H/R treatment (n=6 supernatant samples). (H) Immunostaining of GFP in cas9-tdTomato mouse hearts after injection with Adv-sg-CaSR-GFP (n=6). (I) CaSR mRNA levels in cas9-tdTomato mouse hearts after injection with Adv-sg-NC and Adv-sg-CaSR (n=6). (J) CaSR protein levels in cas9-tdTomato mouse hearts after injection with Adv-sg-NC and Adv-sg-CaSR (n=6). Statistical significance was calculated using an unpaired t-test in H-J, a one-way ANOVA in C-G; *P<0.05, **P<0.01, and ****P<0.0001.

**
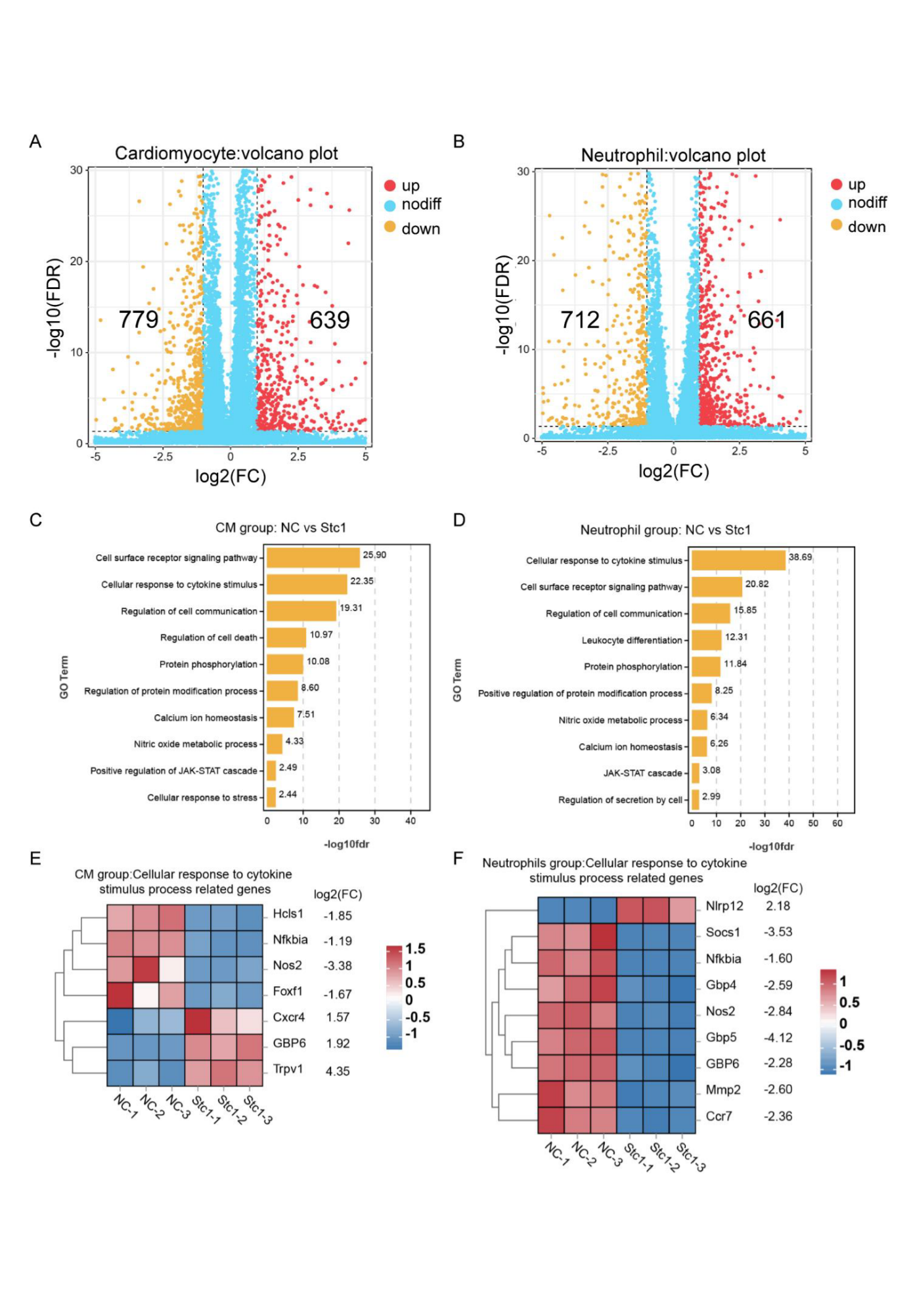
**

**Figure S5. NOS2 was downstream of Stc1/CaSR signaling that induced cytoprotective effects on cardiomyocytes and promoted N1- to N2- like phenotypic switching.**

(A-B) Volcano map of differentially expressed genes in cardiomyocytes and neutrophils after rStc1 treatment. (C-D) Gene Ontology (GO) enrichment analysis of cardiomyocytes and neutrophils after rStc1 treatment. (E-F) Heat map showing the differentially expressed genes of the cellular response to cytokine stimulus pathways in cardiomyocytes and neutrophils separately after rStc1 treatment.

**
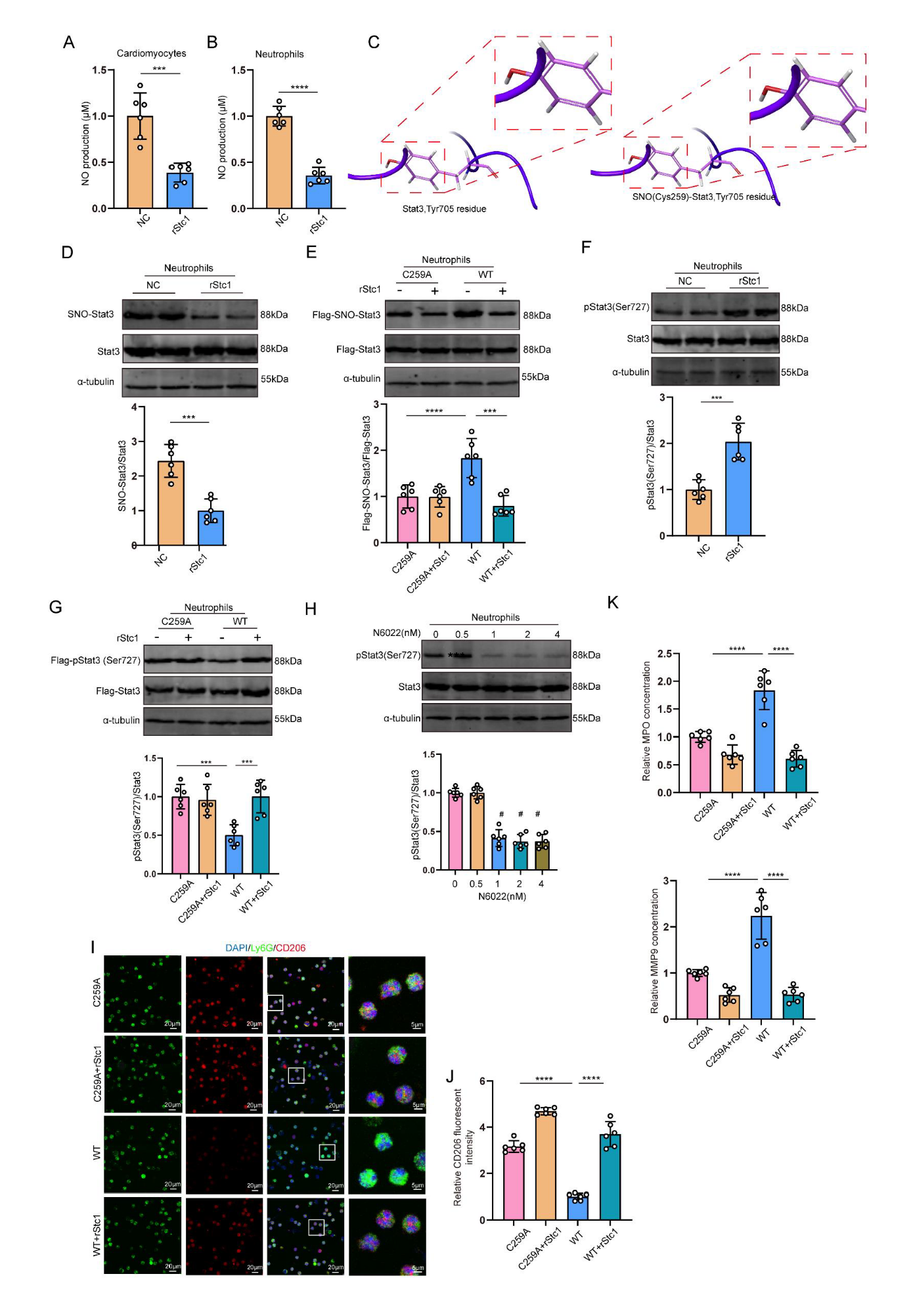
**

**Figure S6. The neutrophil phenotypic switching effect of Stc1 was dependent on NOS2 induced S-nitrosylation of Stat3.**

(A-B) NO detection in cardiomyocytes and neutrophils with and without rStc1, respectively (n=6 cell samples). (C) Structural modeling of Stat3 Tyr705 residue following S-nitrosylation at Cys259. (D) SNO-Stat3 protein levels in NC and rStc1 group neutrophils after H/R treatment (n=6 cell samples). (E) SNO-Stat3 protein levels in neutrophils transfected with Flag-tagged wild-type Stat3 (WT) or its C259A mutant and treated with rStc1 after H/R intervention (n=6 cell samples). (F) pStat3(Ser727) protein levels in NC and rStc1 group neutrophils after H/R treatment (n=6 cell samples). (G) pStat3(Ser727) protein levels in neutrophils transfected with Flag-tagged wild-type Stat3 (WT) or its C259A mutant and treated with rStc1 after H/R intervention (n=6 cell samples). (H) pStat3(Ser727) protein levels in neutrophils treated with N6022 after H/R treatment (n=6 cell samples). (I-J) C259A, C259A+rStc1, WT or WT+rStc1 group neutrophils were collected for immunofluorescent staining for Ly6G (green) and CD206 (red) after H/R treatment (143 neutrophils from 6 mice in the C259A group, 137 neutrophils from 6 mice in the C259A+rStc1 group, 167 neutrophils from 6 mice in the WT group and 148 neutrophils from 6 mice in the WT+rStc1 group; scale bar =20 μm [three on the left], and =5μm [rightmost]). (K) ELISA results showing concentrations of MPO and MMP9 in C259A, C259A+rStc1, WT or WT+rStc1 group neutrophil supernatants after H/R treatment (n=6 supernatant samples). Statistical significance was calculated using an unpaired t-test in A-B, D, F and one-way ANOVA in E, G, H-K; ***P<0.001, and ****P<0.0001, #<0.05 compared with other groups.
